# InSite: a computational method for identifying protein-protein interaction binding sites on a proteome-wide scale

**DOI:** 10.1186/gb-2007-8-9-r192

**Published:** 2007-09-14

**Authors:** Haidong Wang, Eran Segal, Asa Ben-Hur, Qian-Ru Li, Marc Vidal, Daphne Koller

**Affiliations:** 1 Computer Science Department, Stanford University, Serra Mall, Stanford, CA 94305, USA; 2 Department of Computer Science and Applied Mathematics, Weizmann Institute of Science, Rehovot 76100, Israel; 3 Computer Science Department, Colorado State University, South Howes Street, Fort Collins, CO 80523, USA; 4 Center for Cancer Systems Biology (CCSB) and Department of Cancer Biology, Dana-Farber Cancer Institute, and Department of Genetics, Harvard Medical School, Binney Street, Boston, MA 02115, USA

## Abstract

We propose InSite, a computational method that integrates high-throughput protein and sequence data to infer the specific binding regions of interacting protein pairs. We compared our predictions with binding sites in Protein Data Bank and found significantly more binding events occur at sites we predicted. Several regions containing disease-causing mutations or cancer polymorphisms in human are predicted to be binding for protein pairs related to the disease, which suggests novel mechanistic hypotheses for several diseases.

## Abstract 2

InSite is a computational method that integrates high-throughput protein and sequence data to infer the specific binding regions of interacting protein pairs.

## Background

Much recent work focuses on generating proteome-wide protein-protein interaction maps for both model organisms and human, using high-throughput biological assays, such as affinity purification [1-4] and yeast two-hybrid [5-10]. However, even the highest-quality interaction map does not directly reveal the mechanism by which two proteins interact. Interactions between proteins arise from physical binding between small regions on the surface of the proteins [11]. By understanding the sites at which binding takes place, we can obtain insights into the mechanisms by which different proteins fulfill their roles. In particular, when mutations alter amino acids in binding sites they can disrupt their interactions, often changing the behavior of the corresponding pathway and leading to a change in phenotype. This mechanism has been associated with several human diseases [12]. Thus, a detailed understanding of the binding sites at which an interaction takes place can provide both scientific insight into the causes of human disease and a starting point for drug and protein design.

We propose an automated method, called InSite (for Interaction Site), for predicting the specific regions where protein-protein interactions take place. InSite assumes no knowledge of the three-dimensional protein structure, nor of the sites at which binding occurs. It takes as input a library of conserved sequence motifs [13,14], a heterogeneous data set of protein-protein interactions, obtained from multiple assays [2,4,9,10,15,16], and any available indirect evidence on protein-protein interactions and motif-motif interactions, such as expression correlation, Gene Ontology (GO) annotation [17], and domain fusion. It integrates these data sets in a principled way and generates predictions in the form of 'Motif *M *on protein *A *binds to protein *B*'. A key difference between InSite and previous methods [18-20] is that InSite makes predictions at the level of individual protein pairs, in a way that takes into consideration the various alternatives for explaining the binding between this particular protein pair. By contrast, other methods predict affinities between motif types; these predictions are independent of the proteins on which the motifs occur. Thus, InSite may give the same motif pair different binding confidences in the context of explaining different protein-protein interactions. To our knowledge, InSite is the first method that does protein specific binding site predictions. This capability allows us to use InSite to understand specific disease-causing mechanisms that may arise from a mutation that disrupts a protein-protein interaction. InSite also provides a novel framework for integrating evidence from multiple assays, some of which are noisy and some of which are indirect. Unlike other methods, our approach uses all available evidence, and does not assume the existence of a large data set of gold positives.

InSite is based on several key assumptions. The first is that protein-protein interactions are induced by interactions between pairs of high-affinity sites on the protein sequences. Second, we assume that most binding sites are covered and characterized by motifs or domains - conserved patterns on protein sequences that recur in many proteins. (For simplicity, we use the word 'motif' to refer to both motifs and domains, except in cases where we wish to refer specifically to domains.) Although an approximation, this assumption is supported in the literature, as interaction sites tend to be more conserved than the rest of the protein surface [21]. These motifs can correspond to any conserved pattern recurring on protein sequences, whether short regions or entire domains (Figure S1 in Additional data file 2). Finally, we assume that the same motifs participate in mediating multiple interactions. Therefore, we can study a motif's binding affinity with other motifs by examining multiple protein-protein interactions that involve the motif.

**Figure 1:**
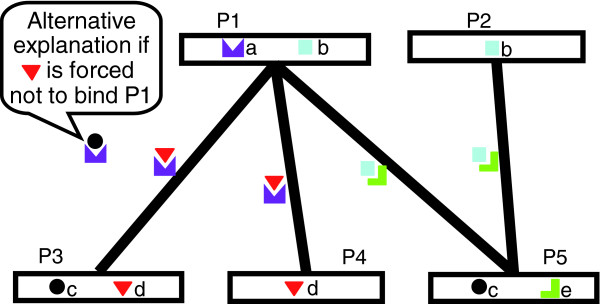
Example illustrating the intuition behind our approach. In this simple example, there are five proteins (elongated rectangles) with four interactions between them (black lines); proteins contain occurrences of sequence motifs (colored small elements within the protein rectangles). Pairs of motifs on two proteins may bind to each other and hence mediate a protein-protein interaction if they have high affinity. The observed interactions are best explained via high affinity for the motif pair *a*,*d*, explaining the interactions *P*_1_-*P*_3 _and *P*_1_-*P*_4_, and high affinity for the pair *b*,*e*, explaining the interactions *P*_1_-*P*_5 _and *P*_2_-*P*_5_. We can now estimate the confidence in a prediction '*P*_*i *_binds to *P*_*j *_at motif *M*' by (computationally) 'disabling' the ability of *M *to mediate this interaction. For example, the prediction that *P*_1_-*P*_4 _bind at motif *d *has high confidence, because *d *is the only motif that can explain the interaction. Conversely, the prediction that *P*_1_-*P*_3 _bind at motif *d *has lower confidence, because the motif pair *a*,*c *can provide an alternative explanation to the interaction. The prediction that *P*_2_-*P*_5 _bind at motif *e *also has high confidence: although interaction via binding at *b*,*c *would explain the interaction, making *b*,*c *a high-affinity motif pair would contradict the fact that *P*_2 _and *P*_3 _do not interact.

InSite is structured in two phases. In the first phase, the algorithm searches for a set of affinity parameters between pairs of motif types that provides a good explanation of the interaction data, roughly speaking: every pair of interacting proteins contains a high-affinity motif pair; non-interacting proteins do not contain such motif pairs; and motif pairs with supporting evidence, such as from domain fusion, should be more likely to have high affinity. There may be multiple assignments to the affinity parameters that explain the data well; our method tends to select sparser explanations, where fewer motif pairs have high affinity, thereby incorporating a natural bias towards simplicity. A simple example of this phase is illustrated in Figure 1; here, the observed interactions are best explained via high affinity for the motif pair *a*,*d*, explaining the interactions *P*_1_*-P*_3 _and *P*_1_*-P*_4_, and high affinity for the pair *b*,*e*, explaining the interactions *P*_1_*-P*_5 _and *P*_2_*-P*_5_. By contrast, the motif pair *c*,*d *is not as good an explanation, because the motif pair also appears in the non-interacting protein pair *P*_3_, *P*_5_. We note that the motif pair *a*,*c *is also a candidate hypothesis, as it predicts the interactions *P*_1_*-P*_3 _and *P*_1_*-P*_5 _and does not incorrectly predict any other interaction. However, it leaves the interaction *P*_1_*-P*_4 _unexplained, therefore leading to a less parsimonious model that also contains the motif pair *a*,*d*.

A set of estimated affinities provides us with a way of predicting, for each pair of proteins, which motif pair is most likely to have produced the binding. In the second phase, we use this ability to produce specific hypotheses of the form 'Motif *M *on protein *A *binds to protein *B*'. In a naïve approach, we can simply take the most likely set of binding sites for the estimated set of affinity parameters. However, in some cases, there may be multiple models that are equally consistent with our observed interaction pattern, but that give rise to different binding predictions. In the second phase of InSite, we therefore assess the confidence in each binding prediction by 'disallowing' the *A*^-^*B *binding at the predicted motif *M*, re-estimating the affinities, and computing the overall score of the resulting model (its ability to explain the observed interactions). The reduction in score relative to our original model is an estimate of our confidence in the prediction. This phase serves two purposes: it increases the robustness of our predictions to noise, and also reduces the confidence in cases where there is an alternative explanation of the interaction using a different motif. For example, in Figure 1, the prediction that 'motif *d *on *P*_4 _binds to *P*_1_' has higher confidence, because *d *is the only motif that can explain the interaction. Conversely, the prediction that 'motif *d *on *P*_3 _binds to *P*_1_' has lower confidence, because the motif pair *a*,*c *can provide an alternative explanation to the interaction. The prediction that 'motif *e *on *P*_5 _binds to *P*_2_' also has high confidence; although interaction via binding at *b*,*c *would explain the interaction, making *b*,*c *a high-affinity motif pair would contradict the fact that *P*_2 _and *P*_3 _do not interact.

**Figure 2:**
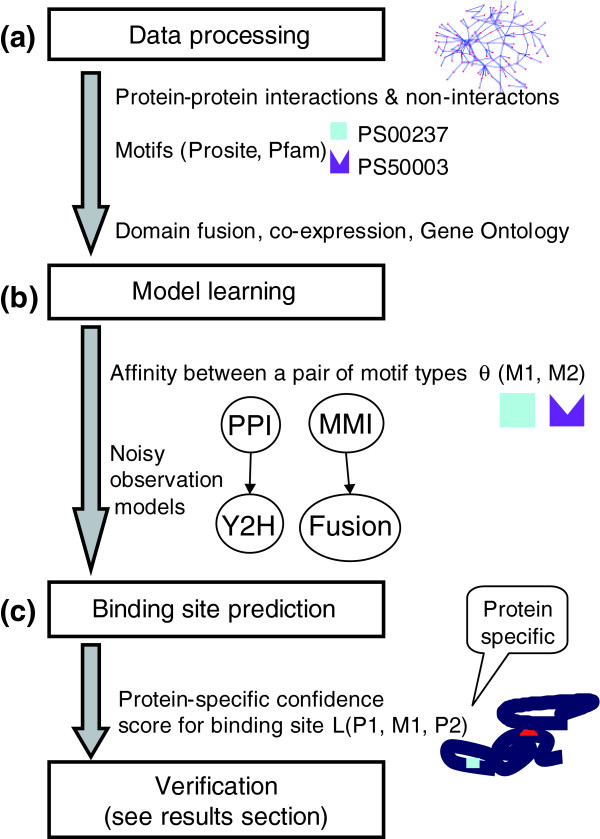
Overview of our automated procedure. Our automated procedure (InSite), which has two main phases, takes as input protein sequences and multiple pieces of evidence on protein-protein interactions and motif-motif interactions. **(a) **Motifs, downloaded from Prosite or Pfam database, were generated based on conservation in protein sequences. Protein-protein interactions are obtained from a variety of assays, including: a small set of 'reliable' interactions, which recurred in multiple experiments or were verified in low-throughput experiments; a set of interactions from yeast two-hybrid (Y2H) assays; and a set of interactions from the co-affinity precipitation assays of Krogan *et al*. [4] and Gavin *et al*. [2]. **(b) **The first phase (Figures S2 and S3 in Additional data file 2) uses a Bayesian network to estimate both the motif pair binding affinities and the parameters governing the evidence models of protein-protein interactions (PPI) and motif-motif interactions (MMI), where the model is trained to maximize the likelihood of the input data. Note that the affinity learnt in this phase depends only on the type of motifs, regardless of which protein pair they occur on. **(c) **In the second phase (Figure S4 in Additional data file 2), we do a protein-specific binding site prediction based on the model learned in the previous phase. For each protein pair, we compute the confidence score for a motif to be the binding site between them. Note that the confidence scores computed here are protein specific and can be different for the same motif depending on the context it appears in.

We provide a formal foundation for this type of intuitive argument within an automated procedure (Figure 2), based on the principled framework of probability theory and Bayesian networks [22]. At a high level, the InSite model contains three components, which are trained together to optimize a single likelihood objective. The first component, inspired by the work of Deng *et al*. [23] and Riley *et al*. [20], formalizes the binding model described above, whereby motif pairs have binding affinities, and an interaction between two protein pairs is induced by binding at some pair of motifs in their sequence. The second and third components, novel to our approach, formulate the evidence models for protein-protein interactions and motif-motif interactions, respectively. They address both the noise in high-throughput assays [24,25], and in the case of protein-protein interactions, the fact that many of the relevant assays are based on affinity purification, which detects protein complexes instead of the pairwise physical interactions that are the basis for inferring direct binding sites. To integrate many assays coherently, InSite uses a naïve Bayes model [24,26,27], where the assays are a 'noisy observation' of an underlying 'true interaction'.

Our entire model is trained using the expectation maximization (EM) algorithm in a unified way (see Materials and methods; Figure S3 in Additional data file 2) to maximize the overall probability of the observed protein-protein interactions. This type of training differs significantly from most previous methods that aggregate multiple assays to produce a unified estimate of protein-protein interactions. These methods [27,28] generally train the parameters of the unified model using only a small set of 'gold positives', typically obtained from the MIPS database [15]. This form of training has the disadvantages of training the parameters on a relatively small set of interactions, and also of potentially biasing the learned parameters towards the type of interactions that were tested in small-scale experiments. By contrast, the use of the EM algorithm allows us to train the model using all of the protein interactions in any data set, increasing the amount of available data by orders of magnitude, and reducing the potential for bias. The same EM algorithm also trains the affinity parameters for the different motif pairs, so as to best explain the observed protein-protein interactions.

These estimated affinities allow us to predict, for each pair of proteins, which motif pair is most likely to have produced the binding. In the second phase, we use these predictions, augmented with a procedure aimed at estimating the confidence in each such prediction, to produce specific hypotheses of the form 'Motif *M *on protein *A *binds to protein *B*'. In this phase, InSite modifies the model so as to enforce that binding between *A *and *B *does not occur at motif *M*. We then compute the loss in the likelihood of the data, and use it as our estimate of the confidence in the binding hypothesis.

As an initial validation of the InSite method, we first show that it provides high-quality predictions of direct physical binding for held-out protein interactions that were not used in training. These integrated predictions, which utilize both binding sites and multiple types of protein-protein interaction data, provide high precision and higher coverage than previous methods. As the primary validation of our approach, we compare the specific binding site predictions made by InSite to the co-crystallized protein pairs in the Protein Data Bank (PDB) [29], whose structures are solved and thus binding sites can be inferred. In our results, 90.0% of the top 50 Pfam-A domains that are predicted to be binding sites are indeed verified by PDB structures. InSite significantly out-performs several state-of-the-art methods: in particular, only 82.0% of the top 50 predictions by Lee *et al*. [19] and 80.0% of the top 50 predictions by Riley *et al*. [20] and of Guimaraes *et al*. [18] are verified in PDB. We also examined the functional ramifications of our predictions. If protein *A *interacts with protein *B *via the motif *M *on *A*, a mutation at motif *M *may have a significant effect on the interaction. If the interaction is critical in some pathway, this mutation may result in a deleterious phenotype, which may lead to disease [30]. We applied InSite to human protein-protein interaction data, and considered those predicted binding motifs *M *that contain a mutation in the Online Mendelian Inheritance in Man (OMIM) human disease database [31] or identified as a potential driver mutation in the recent cancer polymorphism data [32]. We then investigated the hypothesis that the mutation at *M *leads to the disease by disrupting the binding of the protein pair. A literature search validated many of these disease-related predictions, whereas others are unknown but provide plausible hypotheses. Therefore, our predictions provide us with significant insights into the underlying mechanism of the disease processes, which may help future study and drug design.

We have made our predictions and our code publicly available for download [33]. Our algorithm is general, and can be applied to any organism, any protein-protein interaction data set, and any type of motifs or domains.

## Results

### Overview

We applied InSite to data from both *Saccharomyces cerevisiae *and human. For *S. cerevisiae*, we compiled 4,200 reliable protein-protein interactions as our gold standard and 108,924 observations of pairwise protein-protein interactions from high-throughput yeast two-hybrid assays of Ito *et al*. [10] and Uetz *et al*. [9] and assays of Gavin *et al*. [2] and Krogan *et al*. [4] that identify complexes. We also computed expression correlation and GO distance between every pair of proteins, data that have been shown to be useful in predicting protein-protein interactions [34]. Altogether, these measurements involve 4,669 proteins and 82,399 protein pairs. We also constructed a set of fairly reliable non-interactions as our gold standard by selecting 20,000 random protein pairs [35], and eliminating those pairs that appeared in any interaction assay. In the case of human, we used two sets of training data for our analysis. First, we focused on high-confidence pairwise interactions, all of which were modeled as gold positive interactions. These interactions were obtained both from high-quality yeast two-hybrid assays [6] and from the Human Protein Reference Database (HPRD), a resource that contains published protein-protein interactions manually curated from the literature [36]. In the second case, we additionally incorporated into our evidence model the yeast two-hybrid interactions from Stelzl *et al*. [5] and the assay from Ewing *et al*. [37] that identifies complexes. Overall, we obtained 12,411 protein interactions involving 2,926 proteins, and selected 18,745 random pairs as our gold non-interactions, as for yeast.

The InSite method can be applied to any set of sequence motifs. Different sets offer different trade-offs in terms of coverage of binding sites; we can estimate this coverage by comparing residues covered by a particular set of motifs to residues found to be binding sites in some interaction in PDB. One option is Prosite motifs [14], where we excluded non-specific motifs, such as those involved in post-translational modification, which are short and match many proteins. These motifs cover 9.6% of all residues in the protein sequences in our dataset (Figure S1a in Additional data file 2). Of residues that are found to be binding sites in PDB, 37.8% are covered by these Prosite motifs. This enrichment is significant, but many actual binding motifs are omitted in this analysis. An alternative option is to use Pfam domains [38], which cover 73.9% of all the residues; however, PDB binding sites are not enriched in Pfam (Figure S1b in Additional data file 2). Pfam-A domains (Figure S1c in Additional data file 2), which are accurate, human crafted multiple alignments, appear to provide a better compromise: PfamA domains contain only 38.1% of the residues in our dataset, but cover 70.3% of the PDB binding sites. One regimen that seems to work best, which is also used by Riley *et al*., is to train on all Pfam domains (providing a larger training set) and to evaluate the predictions only on the more reliable Pfam-A domains. For each motif set, we used evidence from domain fusion and whether two motifs share a common GO category as noisy indicators for motif-motif interactions [39,40].

We experimented with different data sets and different motif sets. In each case, we trained our algorithm on these data; then, for each interacting protein pair, we compute the binding confidences for all their motifs, and generate a set of binding site predictions, which we rank in order of the computed confidence.

### Predicting physical interactions

The actual protein-protein interactions are mostly unobserved in our probabilistic model. However, we can compute the probability of interaction between two proteins based on our learned model, which integrates evidence on protein-protein interactions and motif-motif interactions as well as the motif composition of the proteins. As a preliminary validation, we first evaluated if InSite is able to identify direct physical interactions. We compare our results to those obtained by using the confidence scores computed by Gavin *et al*. and Krogan *et al*., which are derived from tandem affinity purification (TAP) followed by mass spectrometry (MS) and quantify the propensity of proteins to be in the same complex. Using standard ten-fold cross-validation, we divided our gold interactions and high-throughput interactions into ten sets; for each of ten trials, we hid one set and trained on the remaining nine sets together with our gold non-interactions. We then computed the probability of physical interaction for each protein pair in the hidden set, and ranked them according to their predicted interaction probabilities. We defined a predicted interaction to be true only if it appears in our gold interactions, and false if it appears only in the high-throughput interactions; we then counted the number of true and false predictions in the top pairs, for different thresholds. Although this evaluation may miss some true physical interactions that appear in the high-throughput data set but not in our gold set, it provides an unbiased estimate of our ability to identify direct physical interactions. We separately performed this procedure by ranking the interactions according to the scores computed by Gavin *et al*. and by Krogan *et al*. We also compared our model with a method that combines all evidence on protein-protein interactions in a naïve Bayes model where motifs are not used.

**Figure 3:**
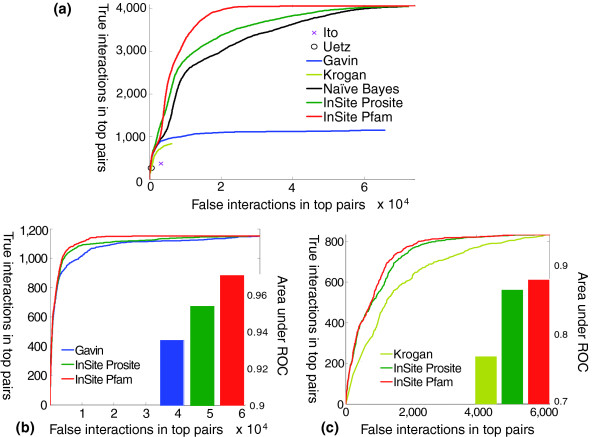
Verification of protein-protein interaction predictions relative to reliable interactions. Protein pairs in the hidden set in a ten-fold cross validation are ranked based on their predicted interaction probabilities (green, red, and black curves for Prosite, Pfam, and naïve Bayes, respectively). Each point corresponds to a different threshold, giving rise to a different number of predicted interactions. The value on the X-axis is the number of pairs not in the reliable interactions but predicted to interact. The value on the Y-axis is the number of reliable interactions that are predicted to interact. The blue and mustard curves (as relevant) are for pairs ranked by Gavin *et al*.'s and Krogan *et al*.'s scores, respectively. **(a) **Predictions for all protein pairs in our data set. As we can see, InSite with Pfam is better than InSite with Prosite, which is in turn better than the naïve Bayes model. All those three models integrate multiple data sets and thus have higher coverage than other methods using a single assay alone. The cross and circle are the accuracies for interacting pairs based on Ito *et al*.'s and Uetz *et al*.'s yeast two-hybrid assays, respectively. **(b) **Predictions only for pairs in Gavin *et al*.'s assay, providing a direct comparison of our predicted probability with Gavin *et al*.'s confidence score on the same set of protein pairs. **(c) **Predictions only for pairs in Krogan *et al*.'s assay, providing a direct comparison of our predicted probability with Krogan *et al*.'s confidence score on the same set of protein pairs.

Our results (Figure 3a) show that InSite is better able to identify direct physical interactions within the top pairs. The area under the receiver operating characteristic (ROC) curve are 0.855 and 0.916 for Prosite and Pfam, respectively, while it is 0.806 for the naïve Bayes model, which integrates different evidence on protein-protein interactions without using any motifs. This shows the motif based formulation is better able to provide higher rankings to the reliable direct interactions (Figure 3a). When comparing with Gavin *et al*.'s and Krogan *et al*.'s scores, our model covers more positive interactions because it integrates multiple assays. However, even if we restrict it only to pairs appearing in a single assay, such as Gavin *et al*.'s or Krogan *et al*.'s, InSite (Figure 3b,c) is able to achieve better accuracy with either Prosite or Pfam. These results illustrate the power of using both an integrated data set and the information present in the sequence motifs in reliably predicting protein-protein interactions. A list of all protein pairs ranked by their interaction probabilities estimated by training on the full data set is available from our website.

### Predicting binding sites

**Figure 4:**
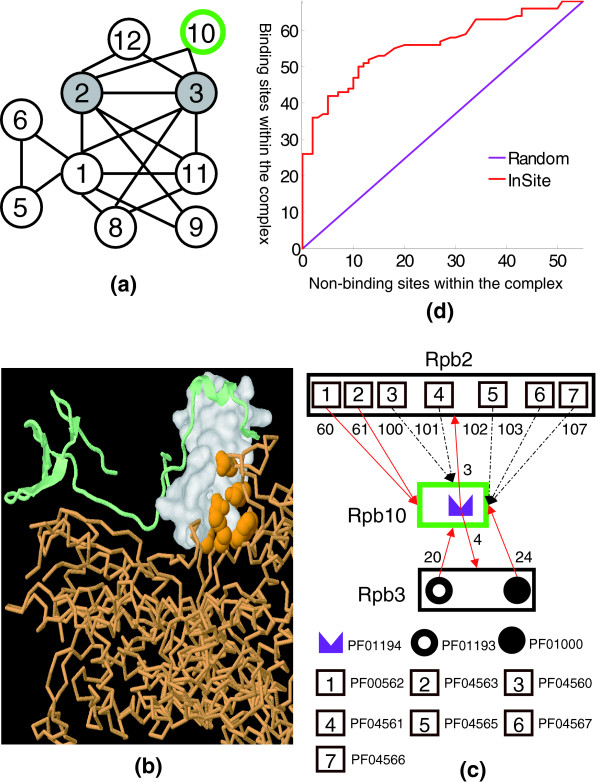
Binding site predictions within the Pol II complex. **(a) **A schematic illustration of interactions within the Pol II complex revealed by its three-dimensional structure. Each circle with number k corresponds to the protein 'Rpbk' (for example, Rpb1). **(b) **One of our top predictions is 'Pfam-A domain PF01096 on Rpb9 binds to Rpb1'. Both Rpb9 and Rpb1 are part of the co-crystallized Pol II complex in PDB (ID: 1I50). Rpb9 is shown as the light green chain with the surface accessible area of the domain rendered in white; Rpb1 is shown as the light orange chain with its residues that are in contact with the domain shown in orange, which verifies our prediction. **(c) **Binding site predictions for interactions involving Rpb10. A red arrow connects a motif to a protein it binds to as revealed by its three-dimensional structure. A dashed black arrow represents a non-binding site. The numbers on the arrow are the ranks based on our predicted binding confidences. We assigned confidence values to a total of 123 motif-protein pairs in this complex. In this case, all six PDB verified binding sites (red arrows) are ranked among the top half, while all five non-binding sites have low confidence values with ranks below 100. **(d) **ROC curve for our motif-protein binding sites predictions within the Pol II complex. There are 123 possible binding sites within the complex that involve the Pfam-A domains in our dataset, out of which 68 (55.3%) are actually binding according to its three-dimensional structure. The possible binding sites are ranked by our predicted binding confidences. The X-axis is the number of non-binding sites within the complex that are predicted to be binding. The Y-axis is the number of PDB verified binding sites that are also predicted to be binding. The purple line is what we expect by chance.

The key feature of InSite is its ability to predict not only that two proteins interact directly, but also the specific region at which they interact. As an example, we considered the RNA polymerase II (Pol II) complex, which is responsible for all mRNA synthesis in eukaryotes. Its three-dimensional structure is solved at 2.8 Å resolution [41], so that its internal structure is well-characterized (Figure 4a,b), allowing for a comparison of our predictions to the actual binding sites. When using Pfam-A domains, the complex gives rise to 123 potential binding site predictions: one for each direct protein interaction in the complex and each motif on each of the two proteins. Among the 123 potential predictions, 68 (55.3%) are actually binding according to the solved three-dimensional structure. We ranked these 123 potential predictions based on our computed binding confidences. All of the top 26 predictions are actually binding (Figure 4d). As one detailed example (Figure 4c), Rpb10 interacts with Rpb2 and Rpb3 through its motif PF01194. We correctly predicted this motif as the binding site for the two proteins (ranked third and fourth). On the other hand, there are nine motifs on the two partner proteins that could be the possible binding sites to Rpb10. Among them, 4 are actually binding, and were all ranked among the top half of the total 123 predictions, while the other 5 non-binding motifs were ranked below the 100th with low confidence scores. Overall, the six binding sites in this example all have higher confidence scores than the five non-binding sites.

We performed this type of binding site evaluation for all of the co-crystallized protein pairs in PDB that also appeared in our set of gold interactions. While the PDB data are scarce, they provide the ultimate evaluation of our predictions. We applied our method separately in two regimens. In the first, we trained on Prosite motifs and evaluated on those motifs that cover less than half of the protein length (Figure S5a in Additional data file 2); we pruned the motif set in this way because short motifs provide us with more information about the binding site location. In the second regimen, we followed the protocol of Riley *et al*., and trained on Pfam domains and evaluated PDB binding sites on the more reliable Pfam-A domains; we also tried to both train and evaluate on Pfam-A domains but the result was worse in comparison to training on all Pfam domains (data not shown).

**Figure 5:**
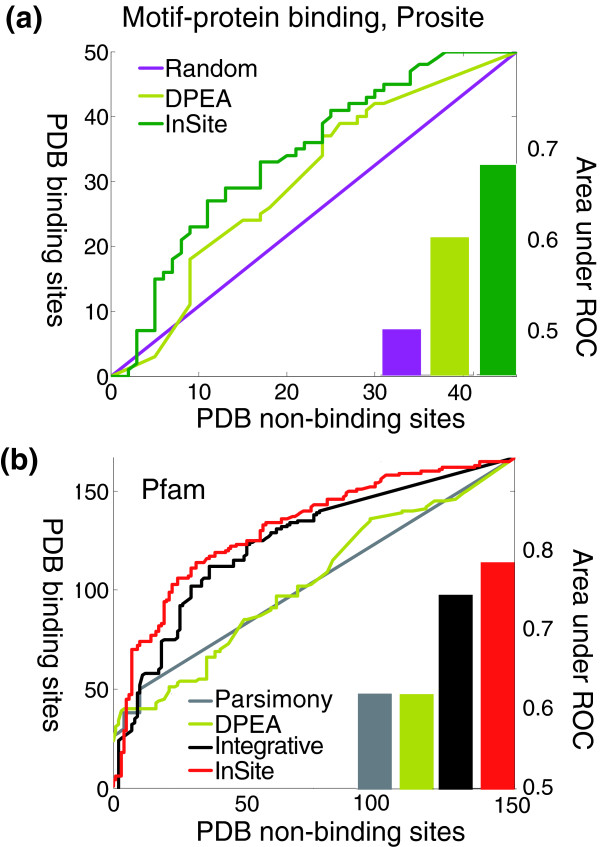
Global verification of binding site predictions. Verification of motif-protein binding site predictions relative to solved PDB structures. Possible binding sites are ranked based on our predicted binding confidences. The X-axis is the number of sites that are non-binding in PDB that are predicted to be binding. The Y-axis is the number of PDB verified binding sites that are also predicted to be binding. The green and red curve are for our InSite with Prosite and Pfam, respectively, which is tailored to binding site prediction and explicitly models the noise in the different experimental assays. The brown curve is for the DPEA score as in Riley *et al*. [20]. The gray curve is for the score derived from the parsimony approach of Guimaraes *et al*. [18]. The black curve is for the integrative approach by Lee *et al*. [19]. The purple curve is what we expect from random predictions. **(a) **Result using Prosite motifs. The area under the curve if we normalize both axes to interval [0,1] are 0.680, 0.601, and 0.5 for InSite, DPEA by Riley *et al*., and random prediction, respectively. **(b) **Result when we train on Pfam domains and evaluate the PDB binding sites only on Pfam-A domains, as in the protocol of Riley *et al*. The area under the curve if we normalize both axes to interval [0,1] are 0.786, 0.745, 0.619, and 0.620 for InSite, integrative approach by Lee *et al*., DPEA by Riley *et al*., and parsimony approach by Guimaraes *et al*., respectively.

Overall, the PDB co-crystallized structures contain 96 potential binding sites covered by Prosite motifs, of which 50 (52.1%) are verified as actually binding, and the remaining 46 are verified to be non-binding. Similarly, PDB contained 317 possible bindings between a Pfam-A domain and a protein, of which 167 (52.7%) are verified in PDB. We ranked all possible bindings according to their predicted binding confidences. With Prosite motifs (Figure 5a), the area under the ROC curve (AUC) is 0.68; note that random predictions are expected to have an AUC of 0.5. For Pfam-A, when trained on all Pfam domains, we achieved an AUC of 0.786 (Figure 5b).

We compared our results to those obtained by the DPEA method of Riley *et al*. [20] the parsimony approach of Guimaraes *et al*. [18], and an integrated approach of Lee *et al*. [19]. DPEA computes confidence scores between two motif types by forcing them to be non-binding, and computing the change of likelihood after reconverging the model with this change. InSite differs from DPEA in two main characteristics: its confidence evaluation method, which is designed to evaluate the likelihood of binding between two particular proteins at a particular site; and the integration of multiple sources of noisy data. Guimaraes *et al*. use linear programming to find the confidence scores to a most parsimonious set of motif pairs that explains the protein-protein interactions. Lee *et al*. use the expected number of motif-motif interactions for a pair of Pfam-A domain types across four species, and integrate them with GO annotation and domain fusion to generate a final ranking on pairs of motif types. Note that all these methods generate confidence scores on pairs of motif types, regardless of what protein pairs they occur on. To use these predictions for the task of estimating specific binding regions, we define the confidence that motif *M *on protein *A *binds to protein *B *as the maximum confidence score between motif type *M *and all the motif types that appear on protein *B*. For Guimaraes *et al*. and Lee *et al*., only the confidence scores between Pfam-A domains are available so we only compared their results with our Pfam-A predictions. We re-implemented DPEA and compared the results with both our Prosite and Pfam-A predictions. As we can see, in both Prosite and Pfam evaluations (Figure 5), the AUC obtained by InSite are the highest (0.786 and 0.680 for Pfam and Prosite, respectively) while Lee *et al*. (0.745 for Pfam only) comes second (Kolmogorov-Smirnov *p *value < 0.0002). InSite is able to reduce the error rate (1 - AUC) by 16.2% compared with Lee *et al*. For Pfam, the AUC values are 0.619 and 0.620 for Riley *et al*. and Guimaraes *et al*., respectively. For Prosite, the AUC value for Riley *et al*. is 0.601. Compared to these two methods, InSite achieves a significant error reduction of 43.7% and 19.8% for Pfam and Prosite, respectively.

If we consider the top 50 predictions made by Insite, 33 (66.0%) are correct for Prosite and 45 (90.0%) are correct for Pfam-A. In comparison, only 52.1% and 52.7% are expected to be correct using random predictions for Prosite and Pfam-A, respectively. The enrichment of known binding sites in our top predictions indicates that InSite is able to distinguish actual binding sites from non-binding sites. In comparison, the proportion of top 50 predictions verified are 82.0% (Pfam-A) for Lee *et al*., 80.0% (Pfam-A) for Guimaraes *et al*., and 80.0% (Pfam-A) and 58.9% (Prosite) for Riley *et al*. Note that, in the case of Pfam-A, Riley *et al*. predicted all top 24 pairs correctly because they are derived from the binding of PF00227 (Proteasome) with itself. This motif pair has the highest score and it appears in 24 binding events, all of which are correctly verified by PDB. The lack of granularity (that is, pairs mediated by the same motif types have the same score) in Riley *et al*. helped in those top predictions, but hurt it in the remaining predictions, thus resulting in overall lower performance.

More generally, a pair of motif types may have multiple occurrences over different protein pairs (Figure S6 in Additional data file 2). The previous methods [18-20] assign the same confidence score to all of them. In order to demonstrate that InSite is able to make different predictions even when both motifs involved are the same, we ran InSite by forcing a pair of motif occurrences between two proteins to be non-binding and used its change of likelihood as a measure of how confident we are about whether these two motifs bind to each other. As an example, transcription factor S-II (PF01096) and RNA polymerase Rpb1 domain 4 (PF05000) are predicted to be more likely to bind when occurring between Rpb9 and Rpo31 than when occurring between Dst1 and Rpo21. This happens because there are fewer motifs on Rpb9 than on Dst1 and the motifs on Rpo31 comprise a subset of motifs on Rpo21. Although some alternative motif pairs between Rpb9 and Rpo31 have high affinity, overall they provide fewer alternative binding sites than those between Dst1 and Rpo21. Furthermore, Rpb9 and Rpo31 are more likely to interact than Dst1 and Rpo21. Therefore, our final confidence score combines the affinity between the two motifs, the presence of other motifs on the proteins, and the interaction probability between the two proteins. Indeed, PDB verifies PF01096 and PF05000 to bind between Rpb9 and Rpo31, but not between Dst1 and Rpo21. The same reasoning applies to binding site predictions between a motif and a protein.

### Understanding disease-causing mutations in human

While a systematic validation is not possible in human, due to the very low coverage of known protein-protein interactions or binding sites, we performed an anecdotal evaluation that focuses on interactions of particular interest for human disease. Many genetic diseases in human have been mapped to a single amino-acid mutation and cataloged in the OMIM database [31]. The exact pathway that leads to the disease is unknown for many of the mutations. As disrupting protein-protein interaction is one way by which a mutation causes disease [30], our binding site predictions can suggest one possible mechanism for such diseases: if a mutation in protein *A *occurs on a motif *M *that is predicted to be the binding site to a protein *B*, and *B *is involved in pathways related to the disease, it is likely that the mutation disrupts the binding and thus leads to the disease. We ran InSite with two different experimental setups: one using only reliable protein-protein interactions, and the other using both reliable and high-throughput protein-protein interactions. Table 1 lists our top ten predictions from each experiment with relevant literature references. As in yeast, we excluded those motifs that cover more than half the length of the protein, so we focused on short motifs that provide us with more information about the binding site. Note that eight predictions are among the top ten in both experiments, showing the robustness of our method when applied to different protein-protein interaction data. A full list of our predictions is available from our website [33].

**Table 1:** Top binding site predictions in human

Protein	Partner	Binding site	OMIM disease	Pubmed
Using only reliable protein-protein interactions				
***PROC***	***PROS1***	***PS01187***	***Protein C deficiency***	***1615482***
***PROC***	***PROS1***	***PS50026***	***Protein C deficiency***	***1615482***
***BAX***	***BCL2L1***	***PS01259***	***Leukemia***	***9531611***
**MMP2**	**BCAN**	**PS00142**	**Winchester syndrome**	**10986281**
**STAT1**	**SRC**	**PS50001**	**STAT1 deficiency**	**9344858**
**VAPB**	**VAMP2**	**PS50202**	**Amyotrophic lateral sclerosis**	**9920726**
**VAPB**	**VAMP1**	**PS50202**	**Amyotrophic lateral sclerosis**	**9920726**
*MMP2*	*BCAN*	*PS00546*	*Multicentric osteolysis*	10986281
PLAU	PLAT	PS50070	Alzheimer disease	7721771
UCHL1	S100A7	PS00140	Parkinson disease	12032852
				
Integrating high-throughput interactions				
***PROC***	***PROS1***	***PS01187***	***Protein C deficiency***	***1615482***
***PROC***	***PROS1***	***PS50026***	***Protein C deficiency***	***1615482***
***BAX***	***BCL2L1***	***PS01259***	***Leukemia***	***9531611***
**MMP2**	**BCAN**	**PS00142**	**Winchester syndrome**	**10986281**
**PTPN11**	**TIE1**	**PS50055**	**Noonan syndrome 1**	**10949653**
**VAPB**	**VAMP2**	**PS50202**	**Amyotrophic lateral sclerosis**	**9920726**
*MMP2*	*BCAN*	*PS00546*	*Multicentric osteolysis*	10986281
*EFNB1*	*SRC*	*PS01299*	*Craniofrontonasal syndrome*	8878483
PLAU	PLAT	PS50070	Alzheimer disease	7721771
UCHL1	S100A7	PS00140	Parkinson disease	12032852

We list the top 10 binding site predictions in human that contain disease causing mutations. The top part lists the predictions when using only reliable protein-protein interactions. The bottom part lists the predictions when integrating high-throughput interactions. Eight predictions appear in both panels, showing our method is robust to the change in the input data. Shown are the protein, its interacting partner, the motif that is predicted to be the binding sites to its partner, the disease caused by the mutations inside the motif, and the Pubmed reference to the interaction. Three of top predictions are verified by literature (in bold and italics), four in the top panel and three in the bottom panel are supported by existing evidence (in bold), one in the top panel and two in the bottom panel are confirmed to be wrong (in italics), and the remaining two predictions do not have literature information. In some cases, it is possible that the mutations at the binding site disrupt the interaction, and thus lead to the disease. *PS01187*, calcium-binding EGF-like domain; *PS50026*, EGF-like domain; *PS01259*, BH3 motif; *PS00142*, metallopeptidase zinc-binding region; *PS50001*, SH2 domain; *PS50055*, PTP type protein phosphatase; *PS50202*, major sperm protein (MSP) domain; *PS00546*, cysteine switch; *PS01299*, ephrins signature; *PS50070*, Kringle domain; *PS00140*, ubiquitin carboxy-terminal hydrolase cysteine active-site.

Some of our predictions are directly validated in the literature. One of the top ten predictions involves vitamin K-dependent protein C precursor PROC, which is predicted to bind to vitamin K-dependent protein S precursor PROS1. There are four regions on PROC, a Gla domain, an EGF-like domain 1, an EGF-like domain 2, and a serine proteases domain. Prosite has ten motifs on the protein, covering these four regions. InSite predicted two of the motifs (PS01187 and PS50026), which correspond to EGF-like domain 1, to be the binding site for PROS. Ohlin *et al*. [42] showed that antibody binding to the region of the EGF-like domain 1 reduces the anticoagulant activity of PROC, apparently by interfering with the interaction between activated protein C and its cofactor PROS1. Therefore, they propose the domain to be the binding site on PROC with PROS, thus validating our prediction. A mutation in the domain causes thromboembolic disease due to protein C deficiency [43], matching the fact that defects in PROS1 are also associated with an increased risk of thrombotic disease (Uniprot:P07225). These facts support a hypothesis in which the mutation on PROC leads to the disease by disrupting the interaction with PROS1.

**Figure 6:**
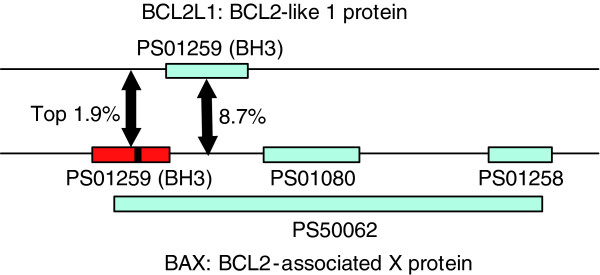
Illustration of human binding site predictions. Schematic representation of our top prediction and its validation by the literature. BAX has four motifs: BH3 motif (PS01259), BH1 (PS01080), BH2 (PS01258), and BCL2-like apoptosis inhibitor family profile (PS50062). BH3 (in red) has the highest change in log-likelihood among those motifs, and is among one of our top predictions (1.9%). Reed *et al*. [48] confirmed that BH3 on BAX is involved in binding with BCL2. On the other hand, the binding site on BCL2 involves portions where all of BH1, BH2, and BH3 reside. Interestingly, none of these motifs on BCL2L1 have high confidence to be a binding site, with the highest one also being BH3 and ranked in the top 8.7%. Mutations in BAX (in position shown by the black bar) cause leukemia.

Another of our highest-confidence binding site predictions is 'the BH3 motif on BAX binds to BCL2L1' (Figure 6). BCL2 has an inhibitory effect on programmed cell death (anti-apoptotic) [44] while BAX is a tumor suppressor that promotes apoptosis. Approximately 21% of lines of human hematopoietic malignancies possessed mutations in BAX, perhaps most commonly in the acute lymphoblastic leukemia subset [45]. There are four motifs on BAX (Figure 6) and we predict BH3 to be the binding site to BCL2 with high confidence (top 1.9%). By searching the literature, we found that Zha *et al*. [46] showed that the BH3 motif on BAX is involved in binding with BCL2, thus validating our binding site prediction. However, BH3 is also required for homo-oligomerization of BAX, which is necessary for the apoptotic function [47]; thus, the BH3 mutation may cause the disease by disrupting the BAX homo-oligemorization. From the BCL2 side, the associated binding site involves the portion where three motifs - BH1, BH2, and BH3 - reside [48]. If we examine the InSite binding site predictions on BCL2, none of the motifs is predicted to have high confidence, with the best one, BH3, ranked at the 8.7th percentile. Therefore, InSite has the flexibility to predict the binding site in one direction, but not the other direction.

Some of our predictions (Table 1) are not directly verified but are consistent with existing literature evidence, and provide biologists with testable hypotheses for possible further investigation. As one example, a mutation at codon 404 in MMP2 causes Winchester syndrome [43]. However, it is not well understood how diminished MMP2 activity leads to the changes observed in the disease [49]. InSite predicted the zinc-binding peptidase region on MMP2, which contains codon 404, to be the binding site to BCAN. As BCAN is degraded by MMP2 [50], the peptidase region we predicted is likely to be the binding site that catalyzes the degradation of BCAN. Codon 404 is believed to be essential for the peptidase activity [43], consistent with our hypothesis that its mutation might disrupt the interaction between MMP2 to BCAN. Our binding site prediction provides one possible hypothesis that implicates BCAN in the process of pathogenesis.

We also listed all top predictions are that are confirmed to be wrong (Table 1). In one case, the prediction involves the Ephrins signature, which is an example of a 'signature motif'. Such motifs represent the most conserved region of a protein family or a longer domain, and are used by Prosite to conveniently identify the longer domain. InSite cannot distinguish the behavior of the signature from the domain. Therefore, when the signature motif is predicted to be the binding site, the actual binding could take place in the longer domain. In the case of the Ephrins signature, Prosite uses the motif to identify the Ephrins protein family. Therefore, we would not generally expect a binding site to overlap the motif.

**Figure 7:**
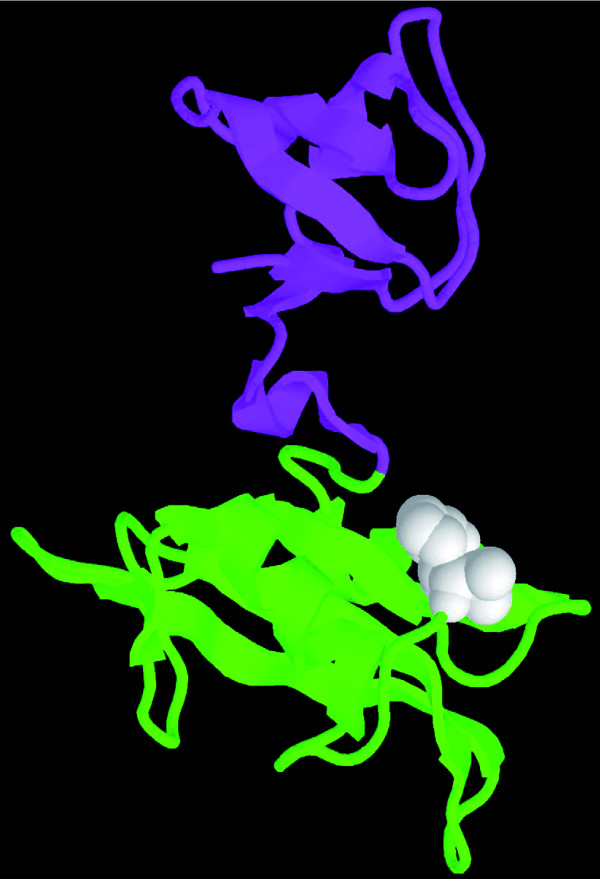
Three-dimensional structure of one of our top predictions. A fragment of FYN with SH2 and SH3 domain is crystallized in PDB (ID: 1G83) and is visualized here. The fragment accounts for about 30% of the total protein length and is rendered in a ribbon representation. The SH2 domain, which is colored in green, is predicted to be the binding site for VAV1. The position of the potential driver mutation found in somatic cancer cells is highlighted by the white balls.

In a similar validation to our OMIM analysis, we considered a recent data set by Greenman *et al*. [32] produced by screening protein kinases for mutations associated with cancer. However, in many cases, it is unknown whether a mutation is a driver mutation that causes the cancer, or whether it is a passenger mutation that occurs by chance in the cancer cell. Even for driver mutations, the mechanism by which it leads to cancer is often unknown. We considered those mutations that fall in InSite predicted binding sites. Among all the potential driver mutations identified by Greenman *et al*., the one most likely to be a binding site according to the InSite predictions is the SH2 domain of FYN in the SRC family (Figure 7), which is predicted to bind to proto-oncogene vav (VAV1). Greenman *et al*. found three mutations on FYN and predicted with 0.985 probability that at least one of them is a driver mutation [32]. This finding suggests the hypothesis that the mutation disrupts the binding of SH2 domain to VAV1, and thus causes cancer. Indeed, a literature search shows that the SH2 domain on FYN is known to bind to VAV1 [51], thereby validating our binding site prediction. Moreover, VAV1 was discovered when DNA from five esophageal carcinomas were tested for their transforming activity [52], which is compatible with the fact that FYN is implicated in squamous cell carcinoma [32]. These observations support the disruption of the FYN-VAV1 binding as the cause for the disease in this case.

## Discussion

Obtaining computational models for the mechanism of protein-protein interactions is an important but challenging task. Other computational methods for discovering protein-protein interaction sites fall into two broad categories. The first are docking methods that try to match two protein structures to find the best sites on both structures [53]. These methods apply only to solved protein structures, which are currently available only for a small number of proteins. To enlarge the set of applicable proteins, some methods [54-57] use homology to proteins with known structures, but many proteins do not, as yet, have any homologues with solved structure, necessitating the use of other techniques. The second class of method uses local sequence information to predict interaction sites [58,59]. These methods typically train a machine learning algorithm (such as a neural network) to identify interaction sites, and, therefore, require solved complexes to provide examples of interaction sites as training data. As such, examples are relatively scarce, the available data might not sufficiently capture the sequence variability found in interaction sites, which can lead these methods to have low sensitivity. Our approach uses only the widely available sequence information and raw protein-protein interaction data, and, therefore, offers the promise of identifying binding sites on a genome-wide scale.

Our approach is most similar to previous work that tries to predict motif-motif or domain-domain interactions. Some of this work focused on best explaining the observed protein-protein interactions [20,23,60-62]. Whereas other methods aim to compute the general affinity between two motif 'types', InSite also explicitly computes the confidence that a specific motif occurrence mediates the binding of a specific interacting protein pair. These finer-grained predictions allow us to identify the specific mechanism for their interaction, whereas other methods that make predictions by looking only at motif types would not be as appropriate for this purpose. For example, the DPEA method by Riley *et al*. [20] computes confidence by forcing two motif types to have affinity 0. In contrast, InSite aims to compute predictions for a specific motif occurrence on an interacting protein pair, and thus forces a particular motif occurrence on a particular protein to be non-binding to another protein. The more global perturbation used by Riley *et al*. would not be as appropriate for this purpose: It may well be the case that a good alternative binding hypothesis exists for the interaction at a particular protein pair, but disallowing all interactions between a pair of motif types causes significant reduction to the likelihood in other protein pairs. Indeed, our method outperforms DPEA, and other state-of-the-art methods like the parsimony approach by Guimaraes *et al*. [18] and the integrative approach by Lee *et al*. [19], at identifying binding regions between an interacting protein pair. Other work [18,19,63,64] infers motif-motif interaction using other types of information, such as co-evolution; this method is shown [64] to generate predictions that have little overlap with DPEA-style methods, and thus can be combined with InSite to gain wider coverage.

InSite is able to integrate different sources of assays in a principled way and learn a different observation model for each assay. It explicitly models the noise from high-throughput assays and the possibility that two proteins in the same complex do not physically interact. This allows us to use the noisy data as well as assays aimed at identifying complexes, so our interaction data set is much bigger than any that have been used before, providing both higher coverage and increased robustness. Our data integration method is unique in not utilizing a 'gold standard' set of interactions (such as ones obtained from low-throughput experiments) for training, thereby greatly increasing the size of the training set and avoiding possible biases in it. InSite also easily accommodates other types of indirect evidence, such as co-expression, GO annotation, and domain fusion, on both protein-protein interactions and motif-motif interactions. This type of integration may be useful in other settings as well. We note that the evidence model, although an important component in our approach, is not the main factor in its performance. Indeed, if we remove the indirect evidence like co-expression, GO annotation, and domain fusion from our model, the AUC value decreases by only 0.033 and 0.019 for Pfam and Prosite, respectively (Figure S7 in Additional data file 2). Therefore, our result using protein-protein interactions alone is still significantly better than the methods of Guimaraes *et al*. and Riley *et al*., which also only rely on protein-protein interaction, and it beats Lee *et al*., which uses multiple types of data, including indirect evidence. On the other hand, if we add our evidence model onto the model of Riley *et al*., the AUC values increase by only 0.017 and 0.009 for Pfam and Prosite, respectively. Therefore, the main component in the performance of our model is the construction of predictions that are targeted at specific protein pairs and take their particular context into account.

There are several limitations to the ability of our approach to identify correct binding sites. Not all motifs mediate protein interactions through direct binding. Some motifs help shape the structure of proteins. Mutations in the motifs would alter the structure of the protein and disrupt binding at some other places. Other motifs are signatures that are markers for longer domains. It is the longer domain, and not the signature motif, that serves as the actual binding site. InSite will not be able to distinguish these cases. One approach would be to classify motifs into either structural or binding motifs by using partially supervised learning with labeled binding sites from PDB or prior biological knowledge. A motif may appear multiple times in a protein, but InSite is unable to distinguish between them, and, therefore, cannot predict which copy is the actual binding site. Most importantly, some binding sites may not be covered by any motif in our set of conserved motifs (Figure S1,5b in Additional data file 2), and thus our current model has no way to predict interactions involving them. Clearly, we can apply InSite to a larger set of motifs, for example, eMotifs [65,66], but there may still be motifs that cannot be identified by conservation. Thus, the most significant extension of our method would be to allow it to search for a motif in cases where there is no pre-existing motif that provides a good explanation for the observed interactions. One possible approach may be an integration of InSite with approaches that use sequence to predict binding sites directly [58,59].

## Conclusion

There has been steady growth in the past few years in the suite of methods that successfully utilize large amounts of available data and sophisticated machine learning methods to solve problems in structural biology for which experimental methods are difficult and time-consuming. These tasks include protein structure prediction [67], RNA structure prediction [68,69], side-chain prediction [70], protein surface prediction, and more. Following in this tradition, we have developed InSite, a novel probabilistic method for predicting regions at which two interacting proteins bind to each other. InSite makes use of three types of data sets: direct protein-protein interaction assays; indirect evidence on protein-protein interactions, such as co-exression; and indirect evidence on motif-motif interactions, such as domain fusion. It provides a principled integration of these data sets, which may be noisy, and may not correspond to direct physical interaction. In future work, the flexibility of the framework would allow us to easily extend it to include more types of information, including structural information. For example, we can use motif-motif binding in PDB to construct a more informed model of the prior distribution for the motif-motif affinity.

InSite makes targeted, testable predictions for specific binding regions in an interacting protein pair. As we have shown, these predictions can be used to generate hypotheses regarding the mechanism by which certain mutations in a protein can disrupt interactions, and give rise to phenotypic changes, including human disease such as cancer. We put all predictions with cancer annotations or OMIM mutations online, allowing for a more comprehensive analysis by experts and follow-on wet-lab experiments. We have also made the InSite software publicly available via the web to allow this tool to be used by researchers. Due to the universal mechanisms underlying biochemical interactions, the tool can be applied to any organism, and even to protein-protein interaction data generated from multiple organisms.

## Materials and methods

### Sources of data

#### *Sccharomyces cerevisiae*

We constructed 'observed interaction' variables for each of the assays, as follows. For the yeast two-hybrid datasets of [9,10], these variables are binary-valued. They take the value 'true' if the pair is observed to interact in the assay, and the value 'false' if both of the two proteins appeared in the assay but the pair was not observed to interact. However, as the number of unobserved interactions grows quadratically in the number of proteins assayed, this procedure would result in too many non-interacting pairs; we therefore keep only those pairs that appeared in some other high-throughput dataset, to allow evidence integration. For the co-AP assays, we selected the interactions with confidence scores above 0.2 from [4] and all interactions from [2], using their confidence scores as continuous observation values. We constructed a 'gold standard' set of *S. cerevisiae *protein-protein interactions from MIPS [15] and DIP [16], downloaded on 21 March 2006. We extracted from MIPS those physical interactions that are non-high-throughput yeast two-hybrid or affinity chromatography. For DIP, we picked non-genetic interactions that are derived from small-scale experiments or verified by multiple experiments. We use this set of reliable interactions as 'gold standard' interactions in our model. For 'gold standard' non-interactions, we picked 20,000 random pairs [35] and removed those that appear in any interaction assays. For these gold standard pairs, we fixed the value of the 'actual interaction' variable accordingly. In all other protein pairs, we leave the actual interaction variables as unobserved. This procedure results in a dataset of 101,065 protein pairs, of which 4,200 were gold standard interactions and 18,666 gold standard non-interactions, and a total of 108,924 observations (Figure S8 in Additional data file 2).

We computed expression correlation using a compendium of time series data obtained in different environmental conditions [71-79]. The compendium has 76 different conditions with a total of 403 time points. For each pair of proteins, we computed the Pearson correlation coefficient across all the time points. We also annotated our proteins with biological processes from GO. For each pair of proteins, we computed the GO distance as the log size of the smallest common category shared by the two proteins. The smaller the value, the more specific category the two proteins belong to, and thus they are more likely to interact [34].

In one run, we used sequence motifs from the Prosite database [14] excluding the non-specific motifs, mostly post-translational modification motifs that appear across many proteins. We removed motifs that are annotated as 'Compositionally biased' or 'DNA or RNA associated'. This gave us 708 different types of motifs with a total of 2,808 motif occurrences. In another run, we used sequence motifs from the Pfam domain database [38], which resulted in 8,089 different types of domains with a total of 11,767 domain occurrences.

We construct a 'domain fusion' variable for each pair of Prosite motifs or Pfam domains. Its value is 1 if the two motifs ever co-occur on the same protein in any species. Its value is 0 otherwise. Note that we use the term 'domain fusion' here, although it can also refer to motifs. We also looked at whether the two motifs appear together in any biological process category based on the mapping table from Pfam to GO [17]. If they do, we assign the 'shared GO' variable to be 1 and we assign it to be 0 otherwise.

#### Human

We used a high confidence yeast two-hybrid assay [6] and HPRD, a resource that contains known protein-protein interactions manually curated from the literature by expert biologists [36] (downloaded on 24 January 2006). The union of these data sets gave us 6,688 reliable interactions. We also used yeast two-hybrid assay from Stelzl *et al*. [5] and an assay that identified co-complex proteins [37] with its confidence score as our observation value. This gave us 5,723 observations. As in yeast, we picked 20,000 random pairs as our gold non-interactions [35] and removed those that appear in any interaction assays. We used the same Prosite motifs, which gave us 687 different types of motifs with a total of 3,034 motif occurrences.

### Learning procedure

#### Probabilistic model

Our probabilistic model has three components. The first (Figure S2 in Additional data file 2, black box) formalizes the binding model described above: for each protein pair in our model, and each pair of motifs on the two proteins, we have a variable indicating whether binding took place at this motif pair. The prior probability that a specific motif pair binds is the affinity of the corresponding motif types. The overall interaction of the proteins is a disjunction of these binding events, and of an additional 'spurious binding' variable that accounts both for noise in some interaction data sets and for binding outside of motifs in our database. The second component of our model (Figure S2 in Additional data file 2, red box) addresses the problem that very few protein interactions are known with certainty. Yeast two-hybrid assays can be noisy [24,25], with a non-trivial fraction of both false positives and false negatives, while affinity purification detects protein complexes instead of the pairwise physical interactions that are the basis for inferring direct binding sites. Moreover, indirect evidence such as co-expression, though useful, only weakly correlates with the actual interactions. Therefore, to integrate many assays coherently, we use a naïve Bayes model [24,26,27]. In this model, we have an 'interaction variable' for each protein pair, whose value is 'true' only when the pair actually interacts. This variable is unobserved in most cases, but serves to aggregate information from a set of partial and noisy assays, which are viewed as 'noisy sensors' for the interaction variable. The quantitative dependencies of these sensors are modeled differently for different assays, to allow for variations in false positive and false negative rate [25,80], and for confidence scores accompanying certain assays [2,4]. There may be multiple observation variables attached to a protein pair, whose interaction probability summarizes the signal from all the assays and is used to learn the binding affinity. The third component of our model (Figure S2 in Additional data file 2, blue box) takes into consideration the noisy evidence on motif-motif interactions. A binding variable between two motifs may have multiple pieces of evidence, all of which serve as noisy sensors for the binding variable and are integrated using a naïve Bayes model in the same way as in the second component.

More formally, each interacting or non-interacting pair of proteins *P*_*i*_, *P*_*j *_is described by an entity *T*_*ij*_. A pair of motifs in two proteins can potentially 'bind' and induce an interaction between the corresponding proteins. We encode this assumption by introducing a variable *T*_*ij*_.*B*_*ab *_for each pair of motifs *a *in *P*_*i *_and *b *in *P*_*j*_, which represents whether the pair of motif occurrences actually binds. The probability that they bind depends on the 'affinity' between the motifs. Therefore, we define:

*P*(*T*_*ij*_.*B*_*ab *_= *true*) = *θ*_*ab*_

and

*P*(*T*_*ij*_.*B*_*ab *_= *false*) = 1 - *θ*_*ab*_

where *θ*_*ab *_is the affinity between motifs *a *and *b*. Note that this affinity is a feature of the motif pair and does not depend on the proteins in which they appear. We place a Dirichlet prior distribution over the value of *θ*_*ab*_, which is the same for *θ *across all motif pairs. We must also account for interactions that are not explained by our set of motifs, such as the binding between amino acids not included in our motif set. Thus, we add a 'spurious binding' variable *T*_*ij*_.*S*. The probability that spurious binding occurs is given by:

*P*(*T*_*ij*_.*S *= *true*) = *θ*_*s*_(*m*) = 1 - (1- *θ*_*s*_)^*m*^

where *m *is proportional to the average (geometrical) number of amino acids not covered by any motif in the two proteins. It represents the fact that the more amino acids we have outside the motif set, the more likely the interaction is induced by something other than binding between motifs. Two proteins interact if and only if some form of binding occurs, whether by a motif pair or by spurious binding. Thus, we define a variable *T*_*ij*_.*I*, which represents whether protein *P*_*i *_interacts with protein *P*_*j*_, to be a deterministic *OR *of all the binding variables *T*_*ij*_.*S *and *T*_*ij*_.*B*_*ab*_. We note that Riley *et al*. did not include a spurious interaction variable in their model, but rather used 0.001, regardless of the protein length, as the probability of interaction when there is no motif pair between two proteins.

To account for the fact that our experimental assays are not direct and reliable measurements of physical protein-protein interactions, we define the observation variables *T*_*ij*_.*O *to be the interactions observed in the experimental assays and indirect evidence like co-expression and GO distance, which are noisy sensors for the actual interaction variable *T*_*ij*_.*I*. Note that an actual interaction variable may have several observation variables if the pair appears in multiple assays. For those assays with binary observations, *T*_*ij*_.*O*_*n *_is a binary variable and the probability it is 'true' depends on *T*_*ij*_.*I *and the type of assay. Therefore, we can account for the different false positive and false negative rates in different assays. For Gavin *et al*., we assume the confidence score *T*_*ij*_.*O*_*g *_to be Gaussian distributions, whose mean and variance depends on the *T*_*ij*_.*I*. For Krogan *et al*., we assume the confidence score *T*_*ij*_.*O*_*k *_has a uniform distribution if *T*_*ij*_.*I *is false (non-interacting) and has an exponential distribution if *T*_*ij*_.*I *is true (interacting). For co-expression, we assume the Pearson correlation coefficient *T*_*ij*_.*O*_*e *_to be Gaussian distributions, whose mean and variance depends on the *T*_*ij*_.*I*. For GO distance, we assume its value *T*_*ij*_.*O*_*o *_to be an exponential distribution when *T*_*ij*_.*I *is false and a mixture of Gaussian and uniform distribution when *T*_*ij*_.*I *is true (interacting). In the case of human confidence score *T*_*ij*_.*O*_*w *_from Ewing *et al*. [37], we use a mixture of Gaussian and indicator functions with different parameters depending on the value of *T*_*ij*_.*I*. Note that each parametric form was selected by examining the empirical distribution and assessing what model would fit it well.

We use *R*_*ab *_to describe a pair of motif *a *and motif *b*. We introduce a variable *R*_*ab*_.*E*_*g *_to represent whether they share the same GO biological process category and another variable *R*_*ab*._*E*_*f *_for whether they appear together in a domain fusion event. Both variables are probabilistically dependent on the binding variable *T*_*ij*_.*B*_*ab *_and serve as its noisy sensors. Note that *R*_*ab *_is the same regardless of which protein pair *T*_*ij *_it appears in. We use different models for domain fusion and GO distance to account for their different correlation with the actual motif-motif interactions. Note that parameters of the evidence models for protein-protein interactions and motif-motif interactions are all learned from the data. Some of the learned values are illustrated in Figure S2 in Additional data file 2.

An instantiation of our probabilistic model is illustrated in Figure S2 in Additional data file 2 and the conditional probabilities involved are summarized below:

$$P(\theta_{ab}=x)=P(\theta_{s}=x)=\frac{1}{B(\alpha ,\beta )}x^{\alpha -1}{\left(1-x\right)}^{\beta -1}$$

*P*(*T*_*ij*_.*B*_*ab *_= *true *| *θ*_*ab *_= *x*) = *x*

*P*(*T*_*ij*_.*S *= *true *| *θ*_*s *_= *x*) = *θ*_*s*_(*m*) = 1 - (1 - *x*)^*m*^

*T*_*ij*_.*I *= *OR*(*T*_*ij*_.*B*,*T*_*ij*_,*S*)

*P*(*T*_*ij*_.*O*_*n *_| *T*_*ij*_.*I*) = *ρ*_*n*_(*T*_*ij*_.*I*)

*P*(*T*_*ij*_.*O*_*g *_| *T*_*ij*_.*I *= *false*) = *N*(*μ*_*g*0_, *σ*_*g*0_^2^)

*P*(*T*_*ij*_.*O*_*g *_| *T*_*ij*_.*I *= *true*) = *N*(*μ*_*g*1_, *σ*_*g*1_^2^)

*P*(*T*_*ij*_.*O*_*k *_| *T*_*ij*_.*I *= *false*) = 1

*P*(*T*_*ij*_.*O*_*k *_| *T*_*ij*_.*I *= *true*) = *λ*_*k *_exp(-*λ*_*k*_(1 - *T*_*ij*_.*O*_*k*_))

*P*(*T*_*ij*_.*O*_*e *_| *T*_*ij*_.*I *= *false*) = *N*(*μ*_*e*0_, *σ*_*e*0_^2^)

*P*(*T*_*ij*_.*O*_*e *_| *T*_*ij*_.*I *= *true*) = *N*(*μ*_*e*1_, *σ*_*e*1_^2^)

*P*(*T*_*ij*_.*O*_*o *_| *T*_*ij*_.*I *= *false*) = *λ*_*o *_exp(-*λ*_*o*_(8.68 - *T*_*ij*_.*O*_*o*_))

*P*(*T*_*ij*_.*O*_*o *_| *T*_*ij*_.*I *= *true*) = *w*_*o*1_*N*(*μ*_*o*1_, *σ*_*o*1_^2^) + *w*_*o*2_*U*(7,8.68)

*P*(*T*_*ij*_.*O*_*w *_| *T*_*ij*_.*I *= *false*) = *w*_*w*1_*N*(*μ*_*w*0_, *σ*_*w*0_^2^) + *w*_*w*2_*I*(*T*_*ij*_.*O*_*w *_= 0) + *w*_*w*3_*I*(*T*_*ij*_.*O*_*w *_= NA)

*P*(*T*_*ij*_.*O*_*w *_| *T*_*ij*_.*I *= *true*) = *w*_*w*4_*N*(*μ*_*w*1_, *σ*_*w*1_^2^) + *w*_*w*5_*I*(*T*_*ij*_.*O*_*w *_= 0) + *w*_*w*6_*I*(*T*_*ij*_.*O*_*w *_= NA)

$$P(T_{ij}.O|T_{ij}.I)=\prod_{T_{ij}.O\in T_{ij}.O}P(T_{ij}.O|T_{ij}.I)$$

*P*(*R*_*ab*_.*E*_*g *_| *T*_*ij*_.*B*_*ab*_) = *σ*_*g*_(*T*_*ij*_.*B*_*ab*_)

*P*(*R*_*ab*_.*E*_*f *_| *T*_*ij*_.*B*_*ab*_) = *σ*_*f*_(*T*_*ij*_.*B*_*ab*_)

*P*(*T*_*ij*_.E | *T*_*ij*_.*B*_*ab*_) = *P*(*R*_*ab*_.*E*_*g *_| *T*_*ij*_.*B*_*ab*_)*P*(*R*_*ab*_.*E*_*f *_| *T*_*ij*_.*B*_*ab*_)

where *α*, *β *are the hyper-parameters in the Dirichlet distribution, 8.68 is the maximum value of the GO distance, *n *enumerates the different type of yeast two-hybrid assays, *O*_*g *_is Gavin *et al*.'s assay, *O*_*k *_is Krogan *et al*.'s assay, *O*_*e *_is co-expression, *O*_*o *_is GO distance, *O*_*w *_is Ewing's confidence score, *E*_*g *_is shared GO motif function, *E*_*f *_is domain fusion, *U*() is uniform distribution, and *I*() is indicator function. The observation parameter vector *η *is the union of *η*_*n*_, *μ*, *σ*, *w*, *λ*, *ρ*, *σ*.

#### Learning

The model defines a joint probability over the entire set of attributes, which is the product of all local conditional probability models shown above. Our learning objective is to find affinities between motifs *θ*, probability of spurious binding *θ*_*s*_, and the parameters for the observation models *η*, which maximize the probability over observed evidence on protein-protein interactions T.O, the partial assignment to the actual interactions T.I, and the observed evidence on motif-motif interactions R.E. Our algorithm uses an iterative procedure based on the EM algorithm to find the local maximum. In the E-step, we compute the conditional probabilities for the binding variables T.B., T.S., and the actual interaction variables T.I, given *θ*, *θ*_*s*_, *η*, T.O, R.E, and use those as the soft assignments to the variables. Define:

$$P(T_{ij}.B_{ab}=true|R_{ab}.E)={\theta^{\prime }}_{ab}=\frac{\theta_{ab}P(R_{ab}.E|T_{ij}.B_{ab}=true)}{P(R_{ab}.E)}$$

to be the binding probability given the evidence on motif-motif interactions, where:

*P*(*R*_*ab*_.E) = *P*(*T*_*ij*_.*B*_*ab *_= *true*)*P*(*R*_*ab*_.E|*T*_*ij*_.*B*_*ab *_= *true*) + (1 - *P*(*T*_*ij*_.*B*_*ab *_= *true*))*P*(*R*_*ab*_.E|*T*_*ij*_.*B*_*ab *_= *false*)

By Bayes' rule, we have:

$$P(T_{ij}.B_{ab}=true|T.O;\theta ,\eta )=\frac{{\theta^{\prime }}_{ab}P(T_{ij}.O|T_{ij}.I=true)}{P(T_{ij}.O)}$$

$$P(T_{ij}.S=true|T.O;\theta ,\eta )=\frac{\theta_{s}(m)P(T_{ij}.O|T_{ij}.I=true)}{P(T_{ij}.O=true)}$$

$$P(T_{ij}.I=true|T.O;\theta ,\eta )=\frac{P(T_{ij}.I=true)P(T_{ij}.O|T_{ij}.I=true)}{P(T_{ij}.O)}$$

where

$$P(T_{ij}.I=true)=1-(1-\theta_{s}(m))\cdot \prod_{T_{ij}.B_{ab}\in T_{ij}.B}\left(1-{\theta^{\prime }}_{ab}\right)$$

*P*(*T*_*ij*_.*O*) = *P*(*T*_*ij*_.*I *= *true*)*P*(*T*_*ij*_.*O*|*T*_*ij*_.*I *= *true*) + (1 - *P*(*T*_*ij*_.*I *= *true*))*P*(*T*_*ij*_.*O*|*T*_*ij*_.*I *= *false*).

In the M-step, we compute relevant expected sufficient statistics using the computed soft marginal probabilities as soft assignments. We use maximum likelihood estimation to re-estimate the parameters *θ*, *θ*_*s*_, *η*. This step can be executed efficiently in closed form, using standard methods, for the parameters *θ*, *η*. To estimate *θ*_*s*_, we need to decompose it into *m *variables and apply EM to this approximate form (see Additional data file 1 for details). We repeat the E-step and M-step until the change of likelihood is less than a threshold. Since, in the next phase, we force each motif-protein pair to be non-binding and compare the change of likelihood *L*_*iaj*_, we have to makes sure the threshold used here for convergence is at least a magnitude smaller than *L*_*iaj*_, so the noise would not overwhelm the signal. Here we set the threshold to be 0.01 in terms of change of log-likelihood. Note that Riley *et al*. used the expected likelihood to test convergence, which does not optimize the joint likelihood and may not always increase over the EM steps.

To estimate the two hyper-parameters, *α*, *β *of the Dirichlet distribution, we used two-fold cross-validation on the PDB data set. In this regimen, we select the hyper-parameters so as to optimize performance on one PDB fold, and evaluate performance on the other fold; thus, no data in the test set were used to estimate any of the parameters or hyper-parameters in the model.

#### Binding confidence estimation

Since we explicitly model the binding events between a pair of motifs and between amino acid pairs outside the motif set, it gives us a way to compute the confidence that a motif on a protein binds to another protein. Here the intuition is that if a motif is non-binding, it is dispensable from the model. We first run our model until convergence. To predict whether motif *a *on protein *i *is the binding site to protein *j*, we force *a *not to bind with any motif on protein *j *(Figure S4 in Additional data file 2). We rerun our algorithm with the above constraint and use the change in likelihood as the confidence score of our prediction, which we denote to be *L*_*iaj*_. A high score indicates that forcing *a *not to be the binding site induces a big change in likelihood and is unfavorable. A low score suggests the binding site is dispensable from the model with competing hypotheses that can explain the observed interactions, and thus the prediction is questionable. Unlike the motif affinities *θ*_*ab *_learned from the previous step, here our confidence score *L*_*iaj *_depends on both proteins *i *and *j *and is different for different proteins.

#### Model initialization

If a motif pair does not appear between any pair of interacting proteins, we set its affinity to be 0, an assignment guaranteed to maximize the joint likelihood; this helps simplify our model structure. We set the initial affinity for the remaining motif pairs based on the frequency with which they appear between interacting protein pairs [81]. The observation parameters *η *for the evidence models are initialized based on empirical counts.

### PDB co-crystallized structure

We extracted all structures from PDB that have at least two co-crystallized chains, and whose chains are nearly identical to *S. cerevisiae *proteins. We define two residues to be in contact if the closest distance between their two respective heavy atoms is less than 5 Å. This definition is similar to that of [59]. A motif is said to bind to a protein if they contain a residue pair that is in contact.

### OMIM

To relate our predictions to mutations that cause human genetic diseases, we extracted the allelic variants from OMIM [31], which describes where the mutations occur and their related diseases. We get a total of 737 mutations covering 131 motifs in 97 proteins of our training data.

### Cancer polymorphism

To relate our predictions to mutations in cancer, we extracted more than 1,000 somatic mutations found in 274 megabases of DNA corresponding to the coding exons of 518 protein kinase genes in 210 diverse human cancers [32]. We focused only on those proteins that are predicted to contain driver mutation. This results in a total of 652 mutations covering 489 motifs in 249 proteins of our training set.

## Abbreviations

AUC, area under the ROC curve; EM, expectation maximization; GO, Gene Ontology; HPRD, Human Protein Reference Database; InSite, Interaction Site; MS, mass spectrometry; OMIM, Online Mendelian Inheritance in Man; PDB, Protein Data Bank; Pol II, RNA polymerase II; ROC, receiver operating characteristic; TAP, tandem affinity purification.

## Authors' contributions

All authors read and approved the final manuscript.

## Additional data files

The following additional data are available with the online version of this paper. Additional data file 1 is the supplementary material on the EM algorithm for the spurious binding variable and captions for supplementary figures. Additional data file 2 is supplementary figures.

## Supplementary Material

Additional data file 1 Figure S1 shows motif coverage of protein sequences compared with coverage of the protein-protein interaction binding sites. Figure S2 is an illustration of the Bayesian network used in the first phase of InSite. Figure S3 is a schematic illustration of our EM-based learning algorithm. Figure S4 is an illustration of the Bayesian network used in the second phase of InSite. Figure S5 shows the number of motifs and their lengths on each protein. Figure S6 shows the number of occurrences for each pair of motif types. Figure S7 evaluates motif-protein binding site predictions with or without indirect evidence relative to solved PDB structures. Figure S8 shows the statistics for each protein-protein interaction dataset. Click here for file

Additional data file 2 Supplementary material on the EM algorithm for the spurious binding variable and captions for supplementary figures Click here for file
