# Supplementary material for: InSite: a computational method for identifying protein-protein interaction binding sites on a proteome-wide scale
Source: Genome Biol. 2007 Sep 14;8(9):R192. doi: 10.1186/gb-2007-8-9-r192 (PMC2375030; doi:10.1186/gb-2007-8-9-r192)
Supplement: Additional data file 1 — Figure S1 shows motif coverage of protein sequences compared with coverage of the protein-protein interaction binding sites. Figure S2 is an illustration of the Bayesian network used in the first phase of InSite. Figure S3 is a schematic illustration of our EM-based learning algorithm. Figure S4 is an illustration of the Bayesian network used in the second phase of InSite. Figure S5 shows the number of motifs and their lengths on each protein. Figure S6 shows the number of occurrences for each pair of motif types. Figure S7 evaluates motif-protein binding site predictions with or without indirect evidence relative to solved PDB structures. Figure S8 shows the statistics for each protein-protein interaction dataset. [file gb-2007-8-9-r192-S1.doc]

Supplementary material for

**InSite: a computational method for identifying protein-protein interaction binding sites on a proteome-wide scale**

The probability that a spurious binding occurs between a protein pair is given by$P(T_{ij}.S=true)=\theta_{s}(T_{ij}.m)=1-(1-\theta_{s})^{T_{ij}.m}$ where *T_ij_.m* is proportional to the average (geometrical) number of amino acids not covered by any motif in the two proteins. This is equivalent to having *m* variables $T_{ij}.s_{k}\text{ }k=1,\ldots,m$, each of which has probability $\theta_{s}$ of being *true*, and $T_{ij}.S$ is the deterministic *OR* of these *m* variables.

To learn $\theta_{s}$, in E-step, we compute the posterior marginal probabilities for $T_{ij}.s_{k}$:

$$P(T_{ij}.s_{k}=true|\mathbf{T}.\mathbf{O;\theta,\eta})=\frac{\theta_{s}P(T_{ij}.\mathbf{O}|T_{ij}.I=true)}{P(T_{ij}.\mathbf{O}=true)}$$

In M-step, we re-estimate $\theta_{s}$ by:

$$\theta_{s}=\frac{\sum_{i,j} \sum_{k=1}^{T_{ij}.m} P(T_{ij}.s_{k}=true|\mathbf{T}.\mathbf{O;\theta,\eta})}{\sum_{i,j} T_{ij}.m}$$

Note that we wouldn’t have a closed-form solution if we don’t decompose $T_{ij}.S$ into $T_{ij}.s_{k}\text{ }k=1,\ldots,m$ and instead try to re-estimate $\theta_{s}$ directly from $P(T_{ij}.S=true|\mathbf{T}.\mathbf{O;\theta,\eta})=\frac{\theta_{s}(T_{ij}.m)P(T_{ij}.\mathbf{O}|T_{ij}.I=true)}{P(T_{ij}.\mathbf{O}=true)}$

**Captions for supplementary figures**

**Supplementary Figure S1:**

Motif (domain) coverage of protein sequences compared with coverage of the protein-protein interaction binding sites in yeast. The outer circle represents all residues in all 4,669 protein sequences we used in our data set and the light blue is the portion that’s covered by our motifs (domains). The inner circle represents residues that are identified to be binding sites in PDB. It only includes the 268 proteins in our data set that are crystallized in PDB and thus whose binding sites we can infer. The dark blue is the portion that is covered by our motifs (domains).

(a) Prosite motifs. 9.6% of the residues in our data set are covered by Prosite motifs, 37.8% of the binding residues in PDB-included proteins are covered by Prosite.

(b) Pfam domains. 73.9% of the residues in our data set are covered by Pfam domains, 70.9% of the binding residues in PDB-included proteins are covered by Pfam.

(c) Pfam-A domains. 38.1% of the residues in our data set are covered by Pfam-A domains, 70.3% of the binding residues in PDB-included proteins are covered by Pfam-A.

**Supplementary Figure S2:**

The first phase of InSite, shown here, uses a Bayesian network to estimate the affinities between motif pairs and evidence models for protein-protein interactions and motif-motif interactions. The Bayesian network is trained so as to maximize the likelihood of the observed protein-protein interaction and motif-motif interaction pattern. An illustrative fragment of the Bayesian network, for the protein pair P1-P3 of **Figure 1**, is shown inside the box. The variable *B* represents the actual binding of a protein pair at a particular motif pair, which is never observed, but is shown to correlate with information such as domain fusion and Gene Ontology. We use them as noisy indicators (*E*), which take binary values – whether two motifs ever appear in the same protein and whether they share the same GO biological process category. The variable *S* represents “spurious” binding, which occurs at a region not represented in our set of motif pairs. An actual interaction between the proteins, represented by the variable *I*, occurs whenever any type of binding occurs. Importantly, not all of the data represent high-reliability physical binding between protein pairs: some datasets could be noisy, and the affinity precipitation assays capture entire complexes. We therefore assign the variable *I* the value true in the training data only if the protein pair is a high-reliability physical interaction and assign the value false if it’s among the randomly picked 20,000 pairs. If the pair occurs in high-throughput assays or has some indirect evidence, it is treated as a noisy indicator (*O*). For the binary interaction assays, this indicator is a binary-valued variable; the protein-complex assays of Gavin and Krogan are associated with a numerical score, and are treated as continuous-valued indicators, whose parametric form was derived by examining the data. Indirect evidence such as expression correlation and Gene Ontology, which is shown to correlate with protein-protein interaction, are also used as noisy indicators. Arrows in the Bayesian network represents the variable downstream is probabilistically dependent on its parent variable. The observed variables are colored in orange and stripes are used for partially observed variables. Both the motif binding affinities and the parameters governing the evidence models are learned together using the expectation maximization algorithm, to maximize the likelihood of the observed data. Some examples of the evidence models learnt are shown in the callout boxes.

**Supplementary Figure S3:**

Schematic illustration of our EM-based learning algorithm. It estimates the motif affinities (**θ**) and parameters in the evidence model (**η**) based on the InSite model illustrated in **Supplementary Figure S2**.

**Supplementary Figure S4:**

In the second phase of InSite, we do protein-specific binding site prediction based on the model we learnt in the previous phase (see **Supplementary Figure S2,3**). For each protein pair, we compute the confidence score for a motif to be the binding site between them. This procedure, illustrated here, estimates the effect, on the model likelihood, of disallowing binding at the predicted motif. For example, to estimate the confidence in the prediction that the *P1-P3* binding takes place at motif *a*, we remove the binding variables (*B*) for motif pairs (*a,* *c*) and (*a,* *d*) and their associated noisy indicators (*E*), thereby preventing *a* from being used for binding. We use the change in likelihood as the confidence in this prediction.

**Supplementary Figure S5:**

(a) For each motif occurrence, we compute the its coverage as the ratio of its length to the length of the protein it occurs on. The x-axis is the bin for the coverage. The y-axis is the number of motif occurrences that fall into this coverage bin. As we can see, most of Prosite motifs cover a small fraction of the protein while Pfam-A domains are usually longer.

(b) For each protein, we compute the fraction of its length that is covered by motif in our data set. The x-axis is the bin for the fraction. The y-axis is the number of proteins that fall into this bin. We exclude those proteins that are not covered by any motif. As we can see, if we use Prosite motifs, most proteins will have majority of their residues not covered by any motif.

(c) For each protein, we count the number of motifs it has in our data set. The x-axis is the number of motifs on a protein. The y-axis is the number of proteins with that many motifs. As we can see, most proteins doesn’t have any Prosite motif in our data set, while most of proteins have at least one Pfam-A domains.

**Supplementary Figure S6:**

Number of motif pair occurrences.

(a) Same pair of motif types may occur between multiple pairs of proteins. Here the x-axis is the number of protein pairs, *n*. The y-axis is the number of Prosite motif pairs that occur between exactly *n* pairs of proteins in our data set.

(b) Same as (a), except this is for Pfam-A domains.

**Supplementary Figure S7:**

Verification of motif-protein binding site predictions relative to solved PDB structures. Possible binding sites are ranked based on our predicted binding confidences. The X-axis is the number of sites that are non-binding in PDB that are predicted to be binding. The Y-axis is the number of PDB verified binding sites that are also predicted to be binding. The green (Prosite) and red (Pfam) curve are for our InSite applied to both protein-protein interaction assays and indirect evidences on protein-protein interactions and motif-motif interactions, such as co-expression, GO distance, and domain fusion.

The blue curve is for InSite applied to only protein-protein interaction assays. The purple curve is what we expect from random predictions.

(a) Result using Prosite motifs. The area under the curve if we normalize both axes to interval [0,1] are 0.680, 0.661, 0.500 for InSite applied to all data, InSite applied only to protein-protein interactions, and random predictions respectively.

(b) Result when we train on Pfam domains and evaluate the PDB binding sites only on Pfam-A domains, as in the protocol of Riley *et al*. The area under the curve if we normalize both axes to interval [0,1] are 0.786, 0.754, and 0.500 for InSite applied to all data, InSite applied only to protein-protein interactions, and random predictions respectively.

**Supplementary Figure S8:**

A total of 101,065 pairs are used, among which 4,200 are reliable interactions and 18,666 are gold non-interactions (see Methods). Each of the remaining pairs is associated with observations from one or more of the four high-throughput assays. The size of the four circles represents the number of pairs in each of the experimental assays. The red slice within each circle represents the overlap with reliable interactions, while the blue slice represents the overlap with gold non-interactions. In Gavin’s and Krogan’s assays, each pair is associated with a confidence score. In Ito’s and Uetz’s assays, we have either observed interacting pairs (3,938 for Ito and 821 for Uetz) or non-interacting pairs, which are between the proteins used in the assay but not identified as interacting. The number on the line between two circles is the number of pairs that overlap between the two assays, with only positive interactions considered in the case of the Ito and Uetz assays. Following is a breakdown of the number of pairs in each assay:

Assay name: Reliable interactions / Other observed interactions / Gold non-interactions / Other observed non-interactions

Gavin: 1157 / 69,140 / N.A. / N.A.

Krogan: 847 / 6,591 / N.A. / N.A.

Ito: 1,542 / 2,396 / 7,362 / 16,343

Uetz: 599 / 222 / 706 / 2,019
