# Supplementary material for: InSite: a computational method for identifying protein-protein interaction binding sites on a proteome-wide scale
Source: Genome Biol. 2007 Sep 14;8(9):R192. doi: 10.1186/gb-2007-8-9-r192 (PMC2375030; doi:10.1186/gb-2007-8-9-r192)
Supplement: Additional data file 2 — Supplementary material on the EM algorithm for the spurious binding variable and captions for supplementary figures [file gb-2007-8-9-r192-S2.pdf]

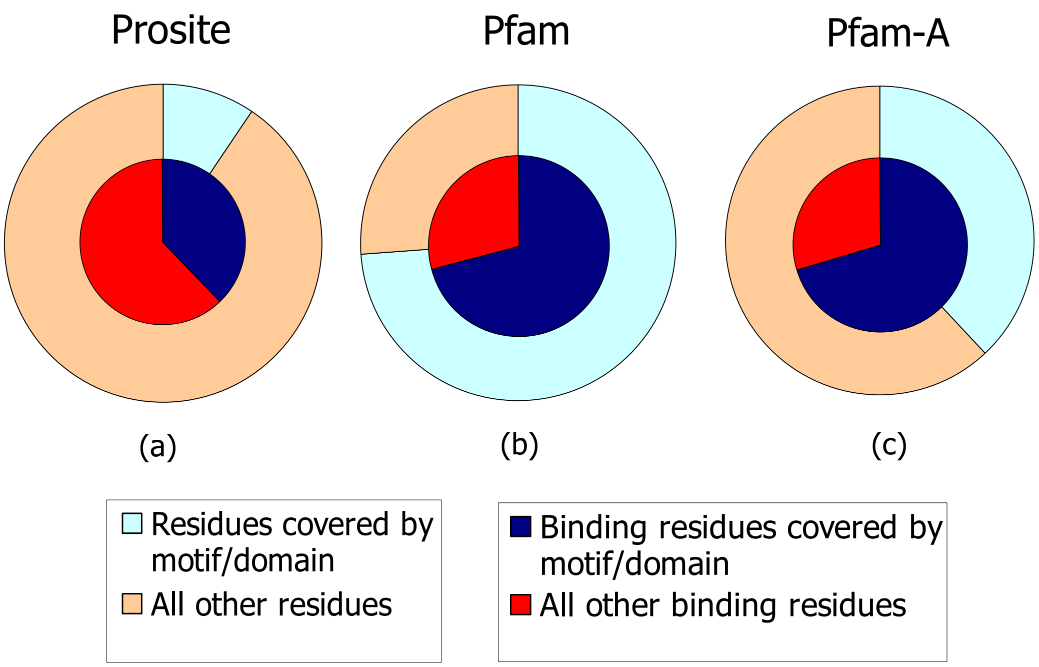

Supplementary Figure S1

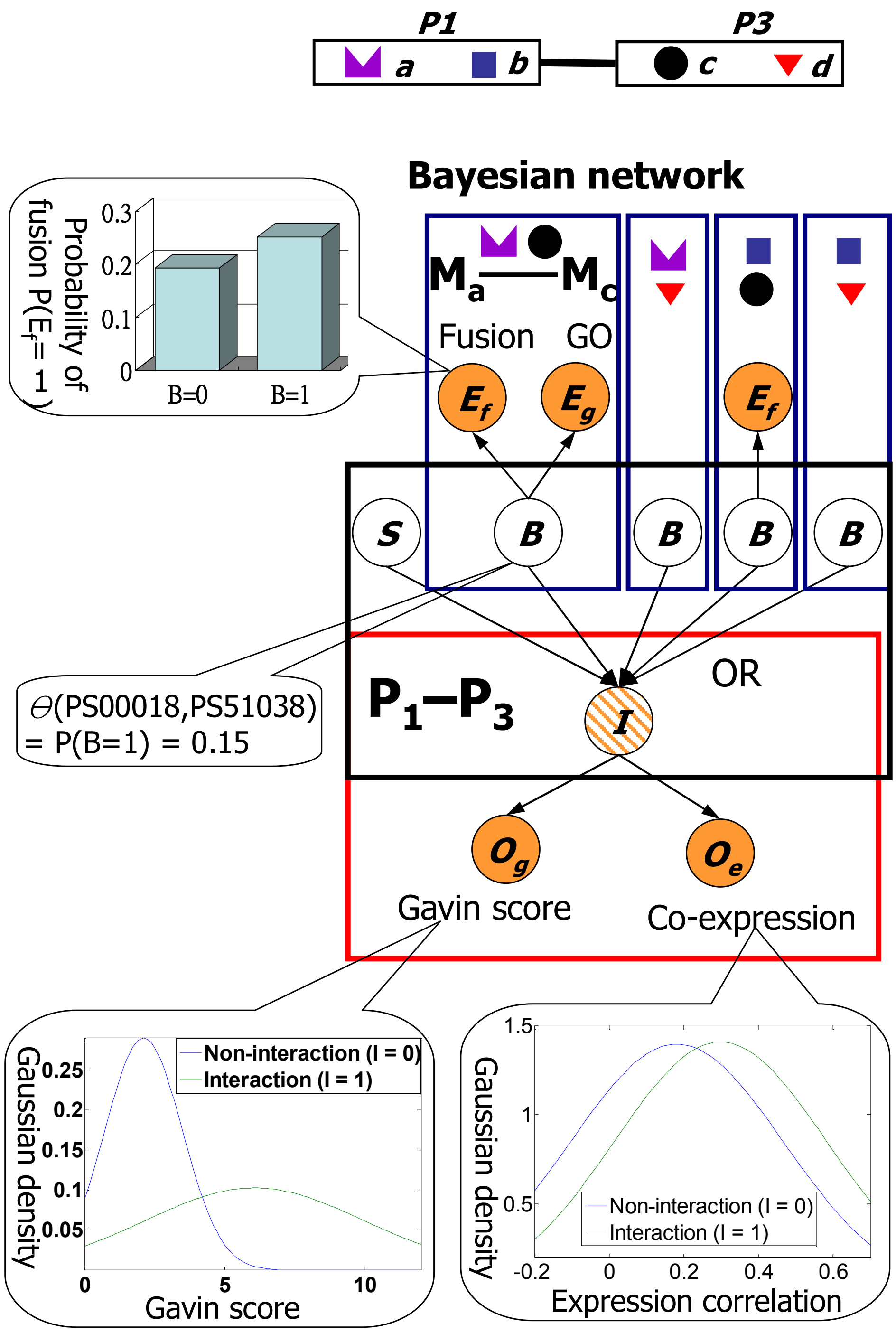

Supplementary Figure S2

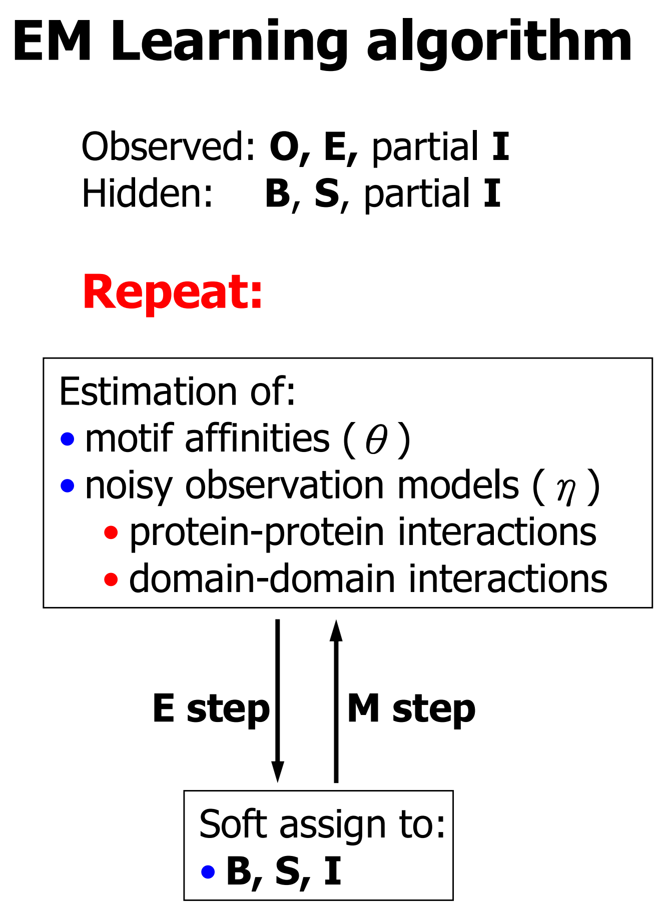

Supplementary Figure S3

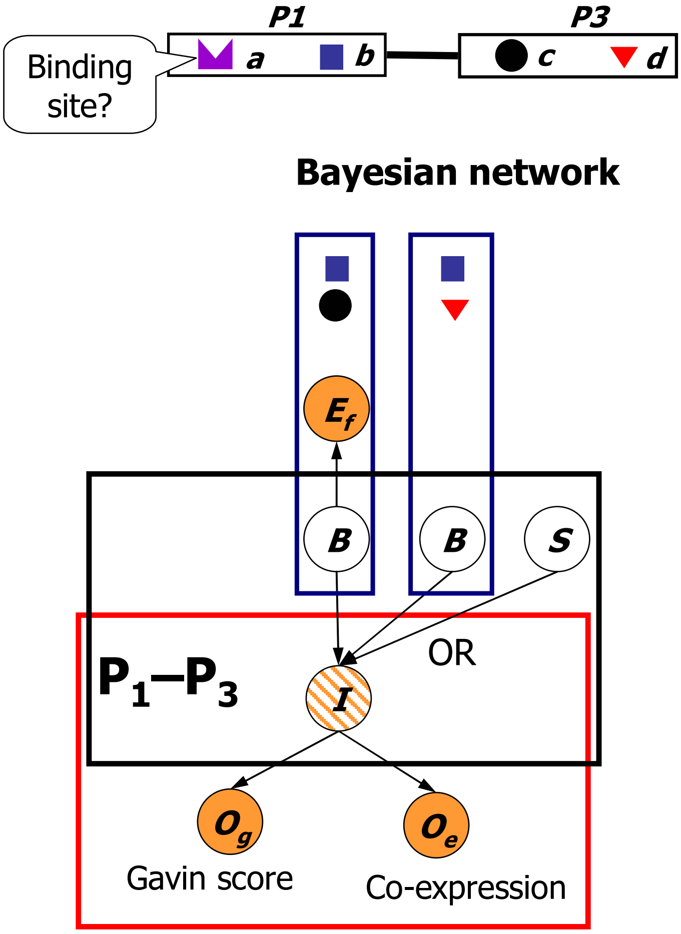

Supplementary Figure S4

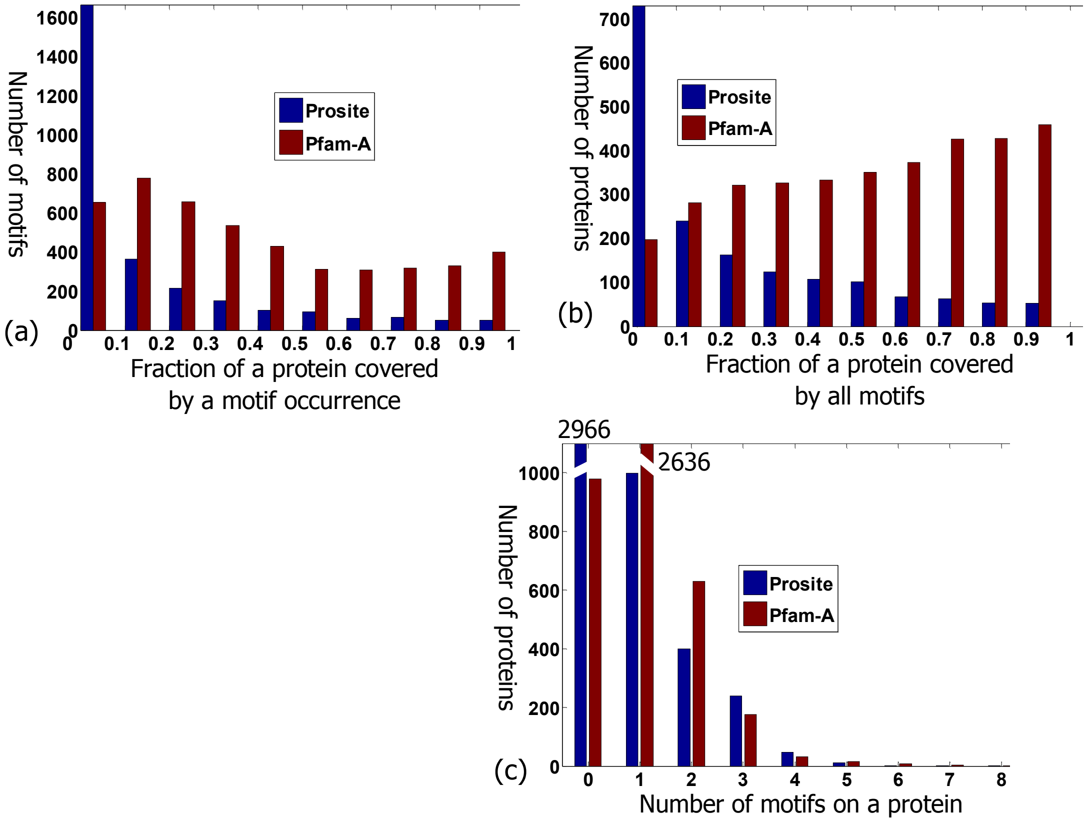

Supplementary Figure S5

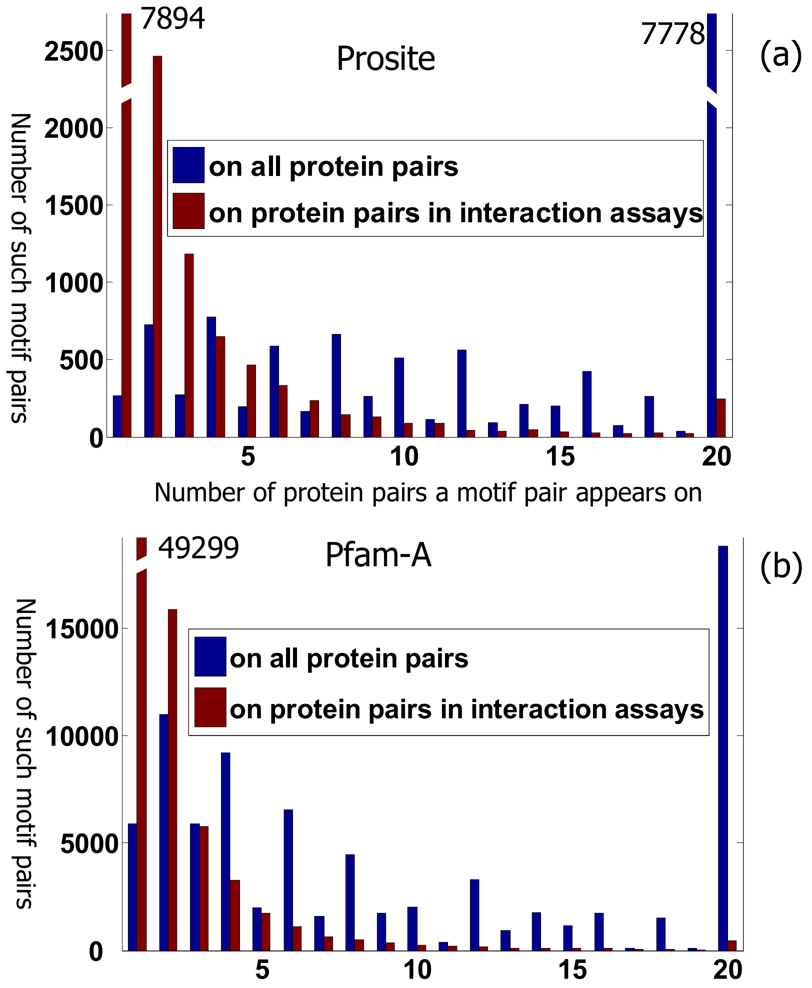

Supplementary Figure S6

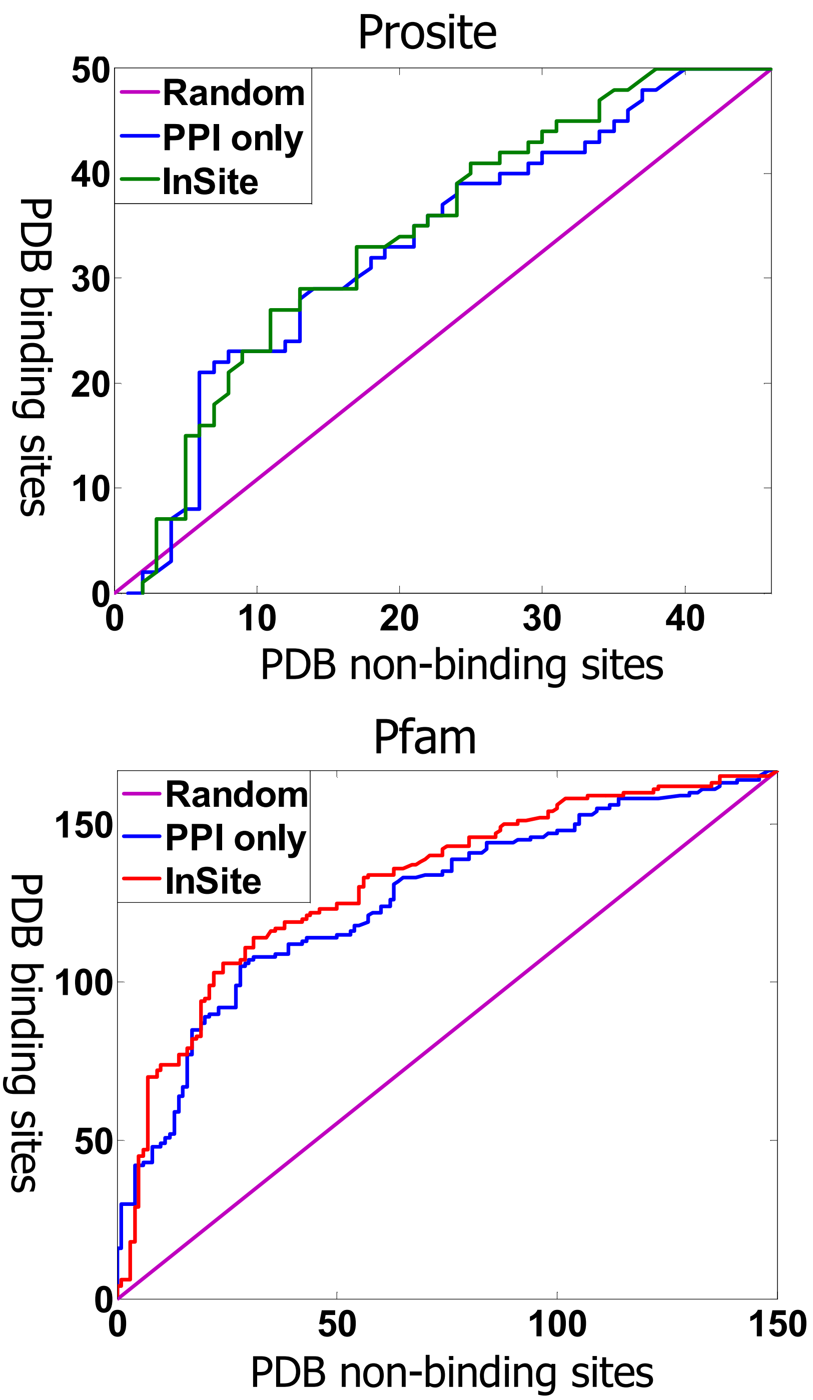

Supplementary Figure S7

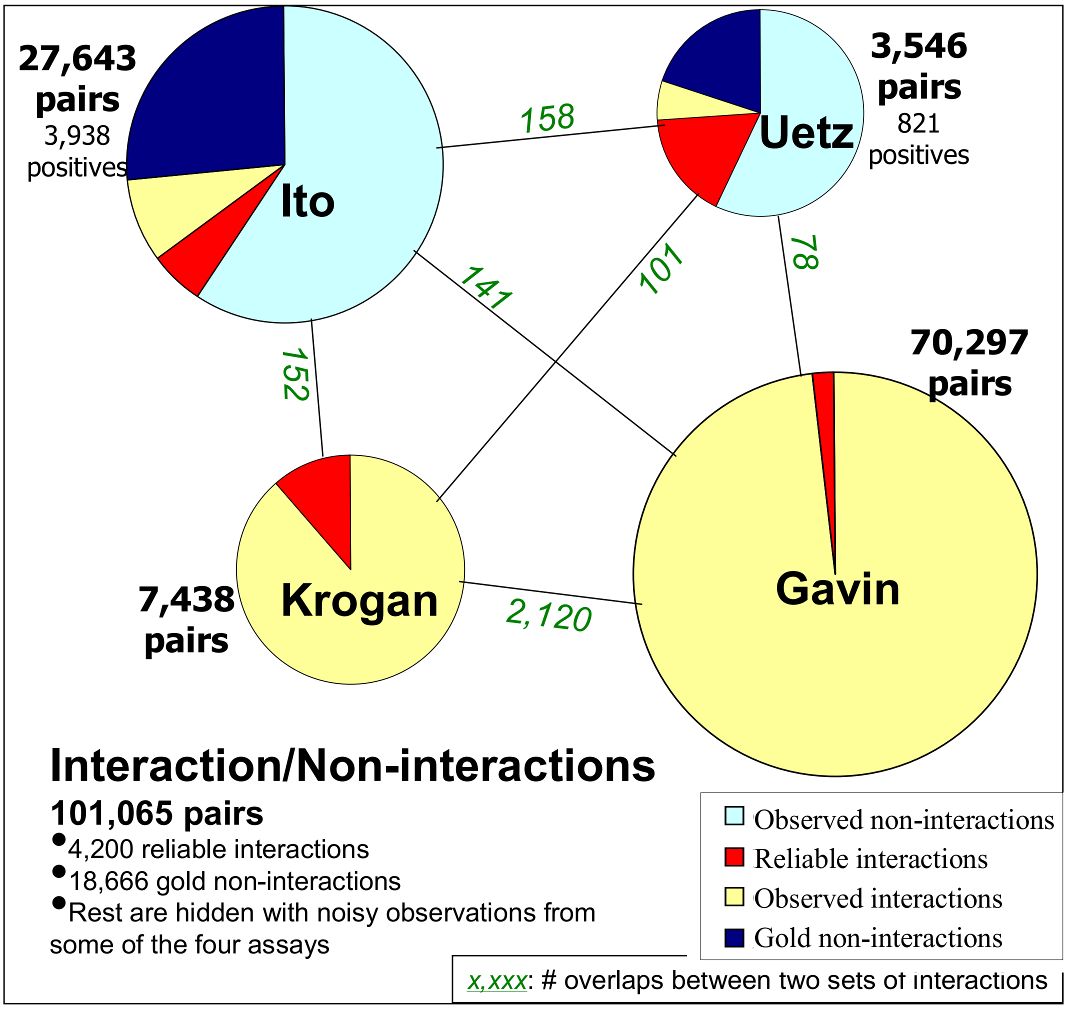

Supplementary Figure S8
